# A network analysis of patient referrals in two district health systems in Tanzania

**DOI:** 10.1093/heapol/czaa138

**Published:** 2020-12-24

**Authors:** Igor Francetic, Fabrizio Tediosi, August Kuwawenaruwa

**Affiliations:** 1 Department of Epidemiology and Public Health, Swiss Tropical and Public Health Institute (Swiss TPH), Socinstrasse 57, 4051 Basel, Switzerland; 2 University of Basel, Petersplatz 1, Basel 4001, Switzerland; 3 Department of Business Economics, Health and Social Care, University of Applied Sciences and Arts of Southern Switzerland (SUPSI), Via Violino 11, Manno 6928, Switzerland; 4 Centre for Primary Care and Health Services Research, University of Manchester, Oxford Road, Manchester M13 9PL, UK; 5 Ifakara Health Institute, Plot 463, Kiko Avenue Mikocheni, Dar es Salaam, Tanzania

**Keywords:** Referral system, networks, child health, non-communicable disease, primary health care

## Abstract

Patient referral systems are fragile and overlooked components of the health system in Tanzania. Our study aims at exploring patient referral networks in two rural districts in Tanzania, Kilolo and Msalala. Firstly, we ask whether secondary-level facilities act as gatekeepers, mediating referrals from primary- to tertiary-level facilities. Secondly, we explore the facility and network-level determinants of patient referrals focusing on treatment of childhood illnesses and non-communicable diseases. We use data collected across all public health facilities in the districts in 2018. To study gatekeeping, we employ descriptive network analysis tools. To explore the determinants of referrals, we use exponential random graph models. In Kilolo, we find a disproportionate share of patients referred directly to the largest hospital due to geographical proximity. In Msalala, small and specialized secondary-level facilities seem to attract more patients. Overall, the results call for policies to increase referrals to secondary facilities avoiding expensive referrals to hospitals, improving timeliness of care and reducing travel-related financial burden for households.

**KEY MESSAGES**

- We study referrals for treatment of childhood illnesses and non-communicable diseases in two rural districts in Tanzania, Kilolo (Iringa region) and Msalala (Shinyanga region).
- In Kilolo, most patients are referred to hospitals due to geographical closeness. There is little role for secondary-level facilities.
- In Msalala, small and specialized secondary facilities receive many referrals. The effect is partially explained by longer travel distance to the closest hospital, compared with Kilolo.
- Referrals to secondary facilities could improve timeliness of care and reduce costs for the health system. Travel-related financial burden for households could also be reduced.

## Introduction

Patient referral systems are crucial, yet weak components of health systems across low- and middle-income countries (LMICs) (Kruk *et al.*, 2018). Such systems rely on a pyramidal structure with local primary facilities covering remote rural communities and disadvantaged urban areas that host the largest share of the population. Primary-level facilities provide simple preventive and treatment procedures with little equipment and human resources capacity, and rely on secondary- and tertiary-level facilities for the treatment and diagnosis of more complicated cases. The referral system should promote the delivery of appropriate healthcare to rural and urban population and contain costs (Hort *et al.*, 2019).

Across sub-Saharan African countries, the effectiveness of the patient referral system is influenced by the transportation infrastructure (Atuoye *et al.*, 2015; Nkurunziza *et al.*, 2016). To this extent, the meagre public budgets available to maintain appropriate roads and means of transportation (e.g. ambulances) undermine the operational efficacy of patient referral arrangements (Hsia *et al.*, 2012). Additionally, the high financial burden associated to private transportation remains a major barrier for successful referral among low-income households (Pembe *et al.*, 2008; Porter *et al.*, 2013; Boex *et al.*, 2015). The referral system in Tanzania faces several challenges including the lack of adequate resources and means to transfer patients (Simba *et al.*, 2008), lack of solid referral criteria and compliance at higher level (Jahn *et al.*, 1998; Jahn and De Brouwere, 2001), poor households’ decision-making process especially for maternal health (Maluka *et al.*, 2020), frequent delays (Schmitz *et al.*, 2019), frequent self-referrals to hospitals related to the perceived poor quality of care at primary-level facilities (Manzi *et al.*, 2012; Yahya and Mohamed, 2018). Yet, to the best of our knowledge, little is known about referral flows, whether dispensaries and health centres effectively act as gatekeepers of the system and which health facility characteristics are associated with the occurrence of referrals between facilities. This study aims to start filling this gap by analysing patient referrals in the two Tanzanian rural districts: Msalala and Kilolo.

### Network analysis and patient referrals

Patient referrals in a given geographical area can be interpreted as networks where each referral represents a directional tie between a pair of health facilities. Network analysis (or social network analysis) is an analytical approach to quantitatively study networks of relations between individuals or organizations, with a broad range of applications in the social sciences (Borgatti *et al.*, 2009). Network analysis should be preferred over other regression techniques to study relational data in light of its ability to deal with interdependent observations, i.e. to account for the existence of ties changing the likelihood of ties to or from adjacent nodes (Pomare *et al.*, 2019). In the last decade, network analysis emerged as a versatile approach to study relationships and networks between healthcare providers (Luke and Harris, 2007; Blanchet and James, 2012). Yet, most published studies addressing connections between healthcare providers are only descriptive (Chambers *et al.*, 2012; Bae *et al.*, 2015) whilst studies addressing the collaboration between healthcare facilities in LMICs remain rare (Sabot *et al.*, 2017).

Studies at the individual level typically analyse complex healthcare situations, mostly in high-income countries. Examples include interpersonal communications during emergency care (Patterson *et al.*, 2013; Hertzberg *et al.*, 2017), collaboration during cardiac implants (Moen *et al.*, 2016), influenza vaccination in teams (Llupià *et al.*, 2016) and medicine prescription process (Boyer *et al.*, 2010; Creswick and Westbrook, 2010; Chan *et al.*, 2017). The studies analysing networks between organizations focus on programme managers implementing development aid projects (Blanchet and James, 2013; Kawonga *et al.*, 2015), healthcare provision organization (Ssengooba *et al.*, 2017; Prusaczyk *et al.*, 2019) and policy-making processes (Shearer *et al.*, 2016, 2014). This latter strand of literature shows a remarkably higher share of studies focusing on LMICs. However, they mostly feature descriptive analyses about observed networks. To the best of our knowledge, there are no published studies in LMICs specifically addressing patient referral systems with network analysis tools. Few authors explored patterns of patient referrals between healthcare organizations in high-income settings, with interesting results (Pallotti *et al.*, 2013; Lomi *et al.*, 2014; Kitts *et al.*, 2017). In light of the above, addressing patient referrals in LMICs from a network analysis angle shall not be interpreted as a methodological musing. Instead, the approach can potentially provide novel insights into the underexplored challenge of strengthening referral systems for primary care.

### Referral system for treatment of childhood illnesses and non-communicable diseases in Tanzania

In this study, we focus on patient referrals related to treatment of childhood illnesses and of some non-communicable diseases (NCDs) in Tanzania. The role of referrals in obstetric and childcare is crucial for the effectiveness of antenatal and postnatal care (Pembe *et al.*, 2010; Hanson *et al.*, 2017). A number of studies report a high risk of child and maternal mortality directly associated to inefficiencies in the referral system (Atuoye *et al.*, 2015; Bohn *et al.*, 2016; Slusher *et al.*, 2018). On the other hand, the increasing burden of NCDs in LMICs, including Tanzania (Kavishe *et al.*, 2015; Kane *et al.*, 2017), is proving taxing on systems designed to deliver primarily maternal and childcare and treatment for infectious diseases (Byass *et al.*, 2014; Kengne and Mayosi, 2014; Mwangome *et al.*, 2017). On the supply side, the treatment of NCDs requires a radical shift to prevention and continuous support for people affected by chronic diseases. To this end, despite policies requiring dispensaries and health centres to be equipped for preventive care and treatment of non-complicated cases related to NCDs, Tanzanian health facilities at primary and secondary level showed poor preparedness (Peck *et al.*, 2014). On the demand side, there is growing evidence of households incurring in catastrophic health expenditures in relation to long-term treatment of chronic and other NCDs, such as cardiovascular diseases and hypertension (Murphy *et al.*, 2020). In response to studies raising such warnings (Peck *et al.*, 2014; Metta *et al.*, 2015; Bintabara and Mpondo, 2018), the Government of Tanzania recently adopted a prevention plan for NCDs (Ministry of Health, Community Development, Gender, Elderly and Children, 2016). The Tanzanian strategy is aligned to WHO guidelines and relies heavily on primary care and prevention as cost-effective tools to curb the growth in health expenditures associated to NCDs (Walley *et al.*, 2012). Notably, access to health services for sick children and NCDs is free of charge in all Tanzanian public health facilities (Mwangome *et al.*, 2017).

The Tanzanian health system is highly decentralized, with districts responsible for budgeting, organization and management of public health facilities (Kigume and Maluka, 2018a). Primary care in Tanzania is delivered mostly through dispensaries, which are evenly spread across the whole country with catchment areas of around 6000 to 10 000 people (Maluka *et al.*, 2018). Secondary care is offered by health centres, facilities that typically have larger catchment areas of about 50 000 people. Health centres are supposed to provide minor surgical care, preventive medicine, some inpatient services and laboratory diagnostics (Hanson *et al.*, 2013). Primary- and secondary-level facilities are mandated to offer preventive care and basic treatment of NCDs. Tertiary care is provided by fully equipped district and regional referral hospitals, and a few national specialized hospitals (Baker *et al.*, 2013). The distribution of facilities across levels of care should reflect the healthcare needs of the population, with the majority of cases treated at the primary level by dispensaries. Slightly more severe cases should be referred to health centres whilst complex cases should be referred to hospitals, generally by health centres and less often by dispensaries, e.g. for emergency conditions (Hanson *et al.*, 2017). To this extent, district hospitals show sign of structural weakness such as underfunding, understaffing and an excessive burden associated to their oversight role over the district healthcare system (McCord *et al.*, 2015). Supplementary Appendix SA1 shows additional details with regard to roles and responsibilities at different healthcare levels within the system. The most recent nation-wide governmental programme for primary health services and referral systems (MMAM) was implemented between 2007and 2017 (Ramsey *et al.*, 2013). With respect to referral systems, the MMAM plan dated 2006 tackled the many known weaknesses, namely a high share of self-referrals to hospitals favoured by understaffing and lack of resources of primary and secondary level, lack of transportation infrastructure and lack of communication facilities to support referrals operations.

To shed light on some of the issues above, this study assessed firstly whether health centres act as effective gatekeepers, mediating referrals from dispensaries to hospitals in two rural districts of Tanzania. Secondly, it explored the health facility characteristics associated with the occurrence of patient referrals.

## Methods

### Data

The data for our analysis were collected in the Tanzanian regions of Iringa and Shinyanga. Although repeated observations over a longer time period would have added value and depth to our study, budget constraints limited our data collection to one round between May and July 2018. Table 1 describes the two regions selected for the study.

**Table 1:** Descriptive statistics about districts

	Kilolo DC	Msalala DC
Region	Iringa region	Shinyanga region
Country zone	Southern highlands	Lake, Northwest
District population (2019)	262 431	323 587
Region population estimate (2019)	1 149 481	1 993 589
Region population density (pers./km^2^)	26	81
Life expectancy at birth (region, years)	44	55
Malaria mortality (region, per 100 000)	21.38	28.67
Under five mortality rate (region, per 1000)	145.1	104.3
Facility deliveries (% of total, 2019)	55.8	78.1
Share of children with reported birth weight below 2.5 kg (2019)	6.5	5.9
Share of caesarean section deliveries (2019)	14.1	2.0
Share of children with pentavalent vaccine at 1 year	89.2	88.6
Full availability of 10 tracer medicines (% of facilities)	96.0	96.5
Health workers density (per 10 000, 2018)	6.3	3.9
Dispensaries		
Public	40	24
Faith-based	15	2
Private	5	3
Health centres		
Public	1	3
Faith-based	1	
Private		1
Hospitals		
Public		
Faith-based	1 (district designated)	
Private		
HPSS project	No	Yes, since 2015

*Notes:* (1) Data from National Bureau of Statistics (2013), MoHCDGEC, NBS, OCGS and ICF International (2016), Malaria Atlas Project (2017) and MOHCDGEC (2020); (2) The list of 10 tracer medicines considered includes: disposable syringe and needles, oral rehydration salts, albendazole/mebendazole oral, amoxycillin/cotrimoxazole, artemether/lumefantrine oral, depo provera, supplies for malaria microscopy, saline solution/dextrose, pentavalent vaccine and oxytocin/ergometrine/misoprostol; (3) Health workers density related to public facilities for the following cadres: nurse, clinical assistant, clinical officer, medical officer, pharmacist and nursing officer; and (4) Msalala district has no public district hospital. The closest district referral hospital is in the neighbouring district of Kahama.

Iringa and Shinyanga are located in different parts of the country and are distant enough to constitute separate referral networks, with no shared referral flow. The data were collected through a survey conducted across all public health facilities and the private facilities officially designated as referral centres across two rural districts: the first, Kilolo in Iringa region, and the second Msalala in Shinyanga region. As of 2019, Kilolo had an estimated population of 262 431; the district is characterized by a mountainous surface, low population density (26 people per square kilometre) and health outcomes well below average. On the other hand, Msalala is slightly more densely populated (population 323 587 and density of 81 people per square kilometre), with a flat highland territory and slightly better health outcomes, although still below national averages. We deliberately focused on patient referral patterns in rural rather than urban districts. Our choice rests on two main arguments: (1) about two thirds of Tanzanians live in rural settlements and; (2) geographical access to care, referral and self-referral is practically much easier in urban areas with higher density of health facilities. The study districts were purposely selected based on an ongoing collaboration with the Health Promotion and System Strengthening (HPSS) project. Since 2015, with the supported of the Swiss agency for Development and Cooperation, HPSS has implemented several activities aimed at strengthening the local health system in the Shinyanga region, hence the selection of Msalala district. Based on contextual knowledge and good connections with neighbouring authorities, HPSS officials recommended the selection of Kilolo district and facilitated the procedures to obtain the required authorizations to conduct research in the area.

The survey focused on the occurrence of patient referral and advice-seeking events related to treatment of childhood illnesses and NCDs in the 3 months prior to the interview. Despite the limited amount of referrals expected over such a short time span, our choice was informed by dialogues with stakeholders and essentially aimed at ensuring higher data quality. Specifically, focusing on patient referrals over the 3 months prior to the interview, we aimed to limit probability of missing data and reduce responder recall bias. We identified respondents within the management team or senior staff of the health facility at the time of the visit. The first part of the questionnaire focused on health facility infrastructure and staffing. In the second part, the respondents were asked to retrieve information concerning the referrals for different conditions from official ledger books at the health facility. For conditions with one or more referrals, respondents were asked additional questions in relation to the referral history of the most recent patient listed in the ledger book. The data collection was carried out using tablet technology based on Open Data Kit (Brunette *et al.*, 2013). Table 2 shows characteristics of the surveyed health facilities and respondents within the facilities. In Kilolo, we collected data from 40 dispensaries, 1 health centre and 1 hospital. In Msalala, we visited 24 dispensaries and 3 health centres. Most of the respondents were in-charge of the health facilities (38.1% and 29.6% in Kilolo and Msalala, respectively) and nurses (45.2% and 55.6%).

**Table 2:** Characteristics of the surveyed sample of facilities and respondents

	Kilolo DC	Msalala DC
Facilities, *N* (%)		
Dispensary	40 (95.2)	24 (88.9)
Health centre	1 (2.4)	3 (11.1)
Hospital	1 (2.4)	0 (0.0)
Total staff assigned (mean, median, range)	5.97, 3, 1–109	6.52, 5, 1–28
Respondent qualification, *N* (%)		
Health facility in-charge (MD, clinician, nurse)	16 (38.1)	8 (29.6)
Clinician or clinical assistant (not in-charge)	3 (7.1)	3 (11.1)
Nurse (not in-charge)	19 (45.2)	15 (55.6)
Midwife (not in-charge)	4 (9.5)	1 (3.7)
Years of tenure (mean, median, range)	5.21, 3.37, 0.3–30	3.20, 3, 0.5–10

The survey collected information regarding several childhood conditions or services identified from the IMCI chart booklet (World Health Organization, 2014), WHO priority areas for childcare (World Health Organization, 2012) and the most prevalent NCDs in Tanzania (Institute for Health Metrics and Evaluation, 2018). Supplementary Appendix SA2 lists and describes the referral conditions addressed by the survey whilst the full questionnaire is available in Supplementary Appendix SA3.

The data cleaning and preparation procedure involved coding the data to binary non-weighted networks. In other words, we imputed an existing referral between a pair of health facilities (i.e. a dyadic tie) for all dyads with an existing referral in one or more categories above. Supplementary Appendix SA4 shows the density of referrals across the categories of care considered.

In spite of 69 health facilities surveyed (42 in Kilolo and 27 in Iringa, see Table 2), the resulting networks of referral flows included a total of 77 network nodes (46 in Kilolo and 31 in Msalala). All referral facilities that are either private or outside of our study districts were not directly surveyed due to budget constraints. However, we were able to obtain a limited amount of contextual data for the full set of facilities in our referral networks (i.e. surveyed referring facilities and non-surveyed referral facilities out of study scope), which we in turn used in the exponential random graph model (ERGM) analyses below. Whilst this limited our inferential analysis—as the set of covariates included in our survey instrument was much richer—we were able to obtain a number of key indicators. These are summarized in Table 3, which describes the distribution of facilities in the referral networks. Facilities managed by private or faith-based organizations do not appear to be important referral points for the surveyed government-managed health facilities. In Kilolo, our network includes only 3 faith-based facilities out of 17 active in the district, as reported in the official health facility registry maintained by the Ministry of Health (see Table 1). In Msalala, one private and one faith-based facility received referrals out of four and two operating in the district, respectively.

**Table 3:** Descriptive statistics for health facilities in the networks

	Kilolo DC (mean, median, range)	Msalala DC (mean, median, range)
Total (*N*)	46	31
Dispensary	40	25
Health centre	3	4
Hospital	3	2
Private health facilities (*N*)	0	1
Faith-based facilities (*N*)	3	1
Population served	29 111, 3321, 1200–950 000	64 471, 10 995, 610–1 535 000
Rooms in the building/compound	5.17, 4, 1–36	4.61, 3, 1–26
Patient beds	21.72, 3, 0–366	24.35, 2, 0–300
Delivery beds	2.022, 1, 0–17	1.64, 1, 0–8
Intensive Care Unit (ICU) beds	0, 0, 0	0, 0, 0
No. of ambulance vehicles	0.26, 0, 0–5	0.35, 0, 0–3
No. of motorcycles	0.26, 0, 0–2	0.26, 0, 0–2
Facility deliveries in the 3 months prior to the survey	65.98, 8.5, 1–1159	125.2, 19, 5–1892
Facility deliveries (per 1000 people served)	9.82, 3.02, 0.40–272.97	7.35, 2.73, 0.32–52.00

Source: MOHCDGEC (2020) and Sawe *et al*. (2014).

Consistently with the large proportion of dispensaries in the sample, the median health facility is rather small and similar in both districts. The average facility infrastructure looks fairly similar in the two districts with about three to four rooms, two or three beds, one delivery bed, no ICU beds and no vehicle. In the analysis, we used the number of facility deliveries based on two considerations. First, a successful facility delivery is likely to increase the chance of subsequent childcare visits to the same health facility (Larson *et al.*, 2014; Kujawski *et al.*, 2015). Second, the number of facility deliveries can be interpreted as an indirect proxy of perceived quality of Reproductive and Child Health (RCH) services at facility level. This latter assumption builds on compelling evidence suggesting a relationship between perceived quality of care and decision to give birth at a specific health facility (Exavery *et al.*, 2014; Tafere *et al.*, 2018).

### Analytical strategy

Our analytical approach is twofold. Firstly, we explored the data with descriptive analysis of network statistics and graphs. Secondly, we analysed factors associated with network and dyad formation using ERGMs.

The descriptive analysis of the observed referral networks concerns structural characteristics of the networks and node-level centrality measures. We used sociograms and maps to visualize data about patient referrals. We then analysed basic structural characteristics of the network, such as the number of nodes (i.e. facilities), edges (referrals) and density. Network density is defined as the number of existing ties out of all possible ties, given the number of nodes in the network (Jackson, 2010). To link our network indicators to volume of healthcare provision, we also report the rate of referrals per 1000 outpatient and RCH cases at referring facilities. At the node level, we analysed the distribution of two specific measures of centrality: in-degree and betweenness. In the network analysis literature, centrality measures are node-level statistics that represent different types of power or popularity in the network (Jackson, 2010). In-degree represents the sum of incoming referrals for each health facility and indicates the extent to which a health facility attracts referrals from other facilities. Technically, betweenness centrality represents the proportion of shortest paths between two nodes that pass through the node of interest (Borgatti *et al.*, 2009). In the context of district health systems, this translates to the proportion of referral paths that link three or more health facilities on which each facility lies on, without being at the extreme ends of the chain. For example, given a dispensary referring a patient to a health centre and the same health centre referring a patient to a hospital, the latter is considered central from the betweenness perspective. Betweenness is the closest centrality concept associated to the gatekeeper role as conceived in the referral system from primary to tertiary care. To assess the extent to which health centres fulfil their gatekeepers’ role, we employed the centrality measures in two alternative ways. First, we visually present the two networks with nodes size proportionate to node-level betweenness. Second, we rank health facilities based on both in-degree and betweenness scores, comparing the resulting ordering.

Visualization and ranking of centrality measures provide a valuable description of the networks and the relative positions of health facilities. To assess the determinants of referrals underpinning the emerging network structure, we employ a set of ERGMs (Robins *et al.*, 2007). ERGMs (also known as p-star models) represent a higher-order analysis of networks that try to model the observed network as a whole. The models measure the contribution of specific characteristic—at network, edge or node level—to the emergence of the specific network observed in the data. The estimation procedure relies on Monte Carlo Markov chains (MCMCs) to approximate a maximum pseudolikelihood estimate for the coefficients contributing to the likelihood of observing the given network, modelled as an exponential probability distribution. The procedure involves comparing the observed network to a large sample of possible random networks (given the number of nodes considered) in order to obtain a maximum-likelihood estimator for the vector of parameters (Van Der Pol, 2019). The main motivation for the development of ERGMs is the inherent inability of regression models to deal with data that exhibit structural dependence, such as ties in a network of collaborating nodes. Similarly to regression models, ERGMs allow testing hypotheses about the role of influence of specific characteristics (at node and edge level) on the formation of ties in the observed network (Byshkin *et al.*, 2018).

Our estimated models include three types of coefficients:

1. Endogenous network structure: edges propensity, isolates propensity, geometrically weighted in-degree distribution.
2. Edge: road distance between the facilities.
3. Facility covariates: type of facility, number of delivery beds, number of patient beds, number of rooms, number of motorcycles, number of ambulances, catchment population and number of facility deliveries (for networks related to treatment of childhood illnesses).

Structural coefficients capture inherent characteristics of the network. The correct parametrization of structural coefficients ensures the convergence of MCMCs. In our case, the most prominent characteristics are low density of the network, high share of isolate nodes (i.e. facilities that do not send or receive referrals) and skewness in the in-degree distribution. Edge and facility covariate try to capture the effect of different covariates on the formation of incoming and outgoing ties, ultimately contributing to the likelihood of the observed network. As discussed above, our selection of covariates was limited to variables that we could obtain for both surveyed and non-surveyed referral facilities (either private or outside of our study districts).

With regard to the analysis of referrals for treatment of childhood illnesses, we applied a stepwise approach. First, we estimated a simple model with only endogenous network and health facility characteristics as explanatory variables (model 1). Second, we added the (log) number of deliveries in the 3 months prior to the survey, which might be a proxy of the level of quality of services (model 2). The rationale for this approach is to assess whether the estimated coefficients change with the inclusion of an output covariate that might be related to quality of service at facility level. For networks of referrals related to treatment of NCDs, we only estimated one model for each district including all endogenous network features and health facility characteristics. Results for all models are reported separately for networks of referrals related to treatment of childhood illnesses and NCDs. The coefficients associated to the explanatory variables represent conditional contributions to log-odds of any given tie. Given a vector of significant model coefficients and the associated vector of change statistics, including the characteristics of a pair of nodes (*i, j*), an inverse-logit transformation returns the probability of observing a tie between nodes *i* and *j*. The intuition can be best understood in the simplest case of a (directed or undirected) network modelled just as a function of the number of edges observed. In this case, an inverse-logit transformation of the ERGM coefficient associated to edges propensity would simply return the observed network density. Put it another way, the probability of a tie between any pair of nodes would depend only upon the total number of edges observed in the network and the number of nodes. Supplementary Appendix SA6 provides a numerical example.

**Figure 1:**
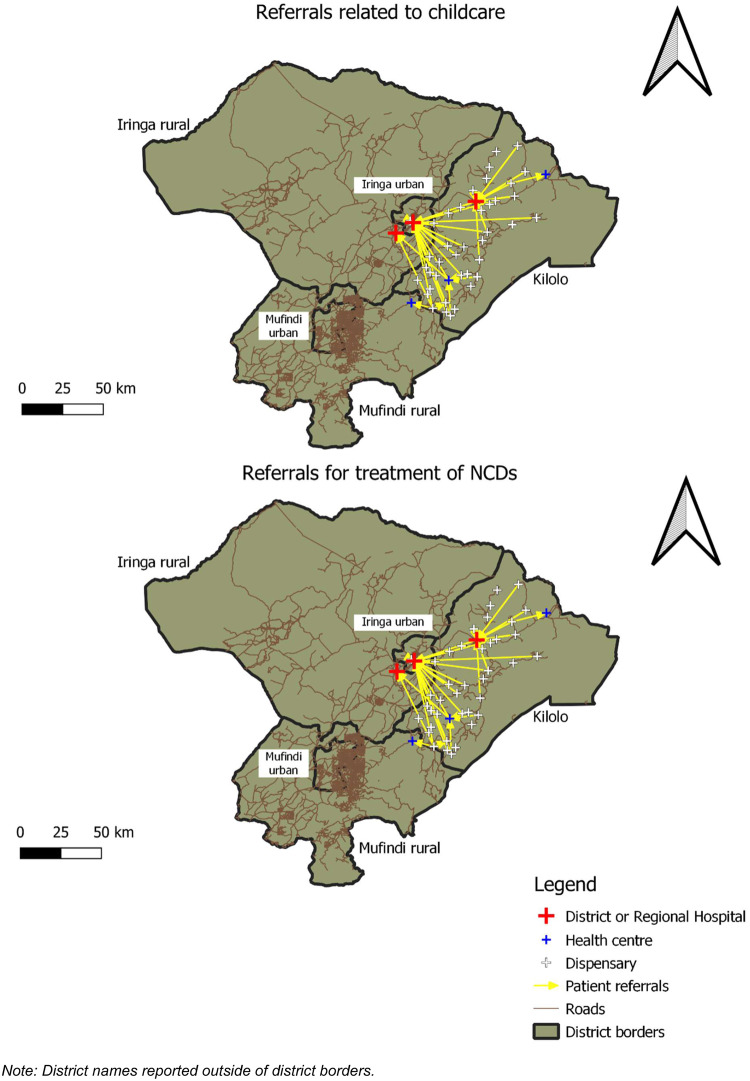
Maps of patient referrals for Kilolo district, Iringa Region. Note: District names reported outside of district borders.

**Figure 2:**
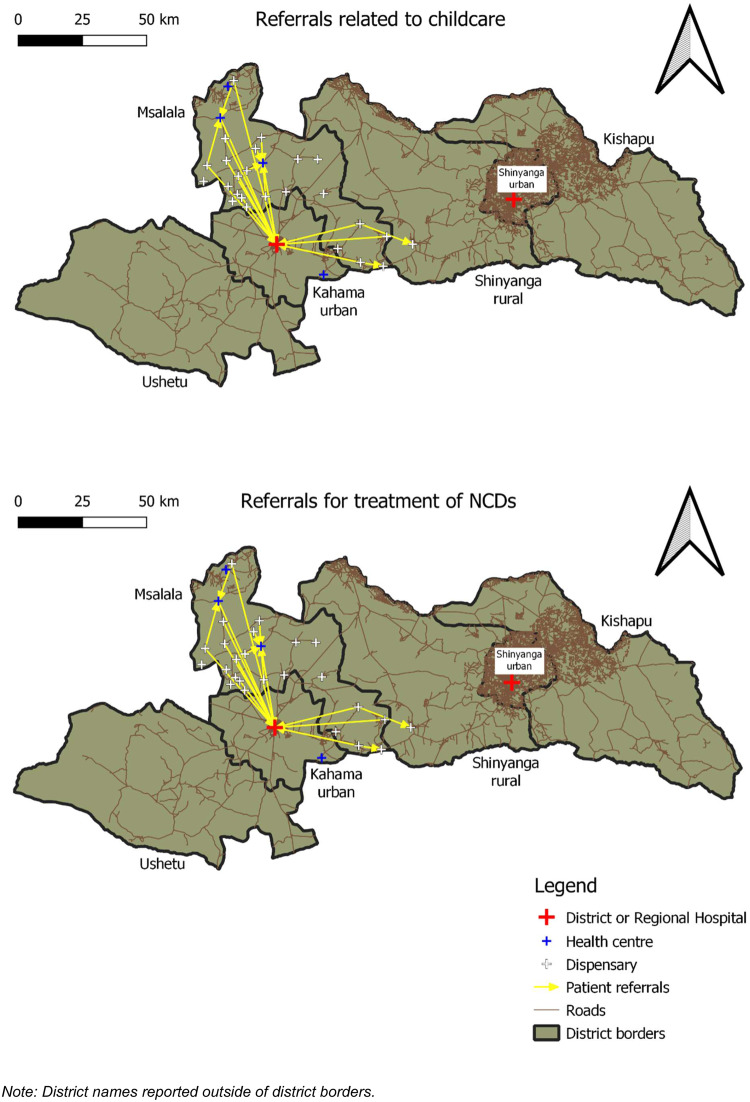
Maps of patient referrals for Msalala district, Shinyanga Region.

**Figure 3:**
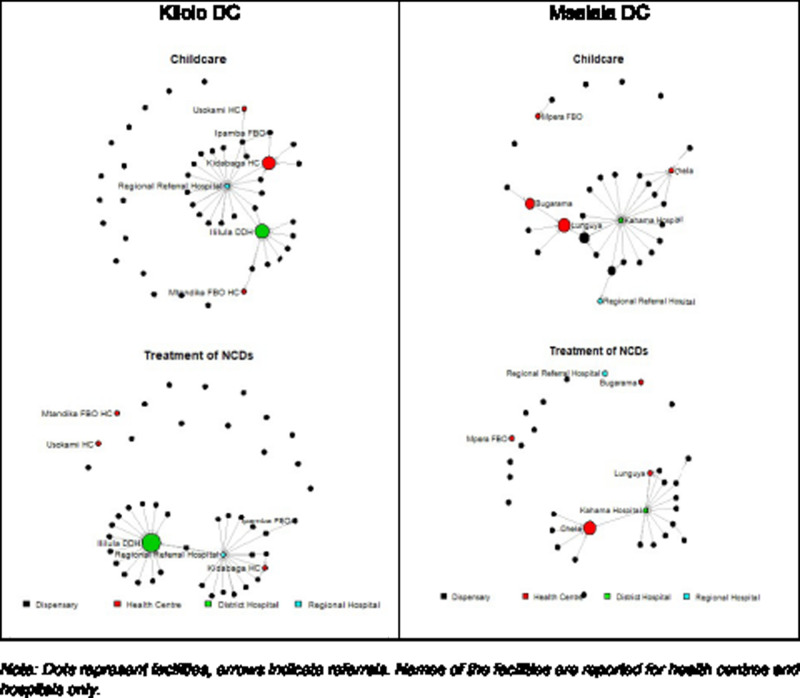
Sociograms for referral networks in Kilolo DC and Msalala DC with nodes size. Note: Dots represent facilities and arrows indicate referrals. Names of the facilities are reported for health centres and hospitals only proportional to betweenness score.

All computations, analyses and sociograms (Figure 3) were obtained using the open-source statistical software R. Maps (Figures 1 and 2) were generated using the open-source GIS software QGIS.

## Results

### Descriptive analysis of referral networks

Table 4 reports basic statistics about the networks of referrals. Most outgoing referrals are from dispensaries towards higher-level facilities. Notably, some referrals are directed from surveyed government health facilities to private facilities. In other cases, referrals are directed from surveyed facilities in the selected districts to public facilities in neighbouring district councils. The referral networks in our analysis show a low density in both districts. The survey confirmed that referrals are rare compared with the overall volume of patient visits. In our sample and over our 3 months study period, 14–24 children were referred to another facility every 1000 RCH clinic visits. Focusing on the treatment of NCDs, only five to seven patients per 10 000 OPD visits were referred.

**Table 4:** Descriptive statistics for networks of referrals related to treatment of childhood illnesses and NCDs

	Kilolo DC	Msalala MC
Treatment of childhood illnesses		
Number of referrals	33	32
Referrals to private or faith-based facilities	9	1
Referrals outside of district boundaries	21	19
Referrals		
Between dispensaries		3
From dispensaries to health centres	5	10
From dispensaries to hospitals	22	16
From health centres to dispensaries	1	
Between health centres		1
From health centres to hospitals	2	2
From district hospital to regional hospital	3	
Network density	0.016	0.034
Referrals per 10 000 outpatient visits	7.34	8.25
Referrals per 1000 RCH visits	24.00	13.69
Treatment of NCDs		
Number of referrals	33	19
Referrals to private or faith-based facilities	15	1
Referrals outside of district boundaries	17	12
Referrals		
Between dispensaries	0	2
From dispensaries to health centres	2	6
From dispensaries to hospitals	25	8
Between health centre		
From health centres to hospitals	1	3
From district hospital to regional hospital	5	
Network density	0.016	0.020
Referrals per 10 000 outpatient visits	7.34	4.89

*Note:* Ratio of inpatient, outpatient and RCH visits based on reported number of visits at the surveyed (referring) health facilities in the 3 months prior to the survey date.

Figures 1 and 2 present the georeferenced maps of referral networks for Kilolo and Msalala districts, respectively. The maps show the entire regions of Iringa and Shinyanga, with district borders highlighted in bold black.

Besides larger extension and a higher number of health facilities in the Kilolo district, the most prominent difference between the two settings is the closeness to the regional capital city. Kilolo district shares border with the Iringa urban district, the extended Iringa town that hosts the regional referral hospital. On the other hand, Msalala is far from Shinyanga town, with parts of the district located >100 km apart from the regional referral hospital in Shinyanga. This difference in geography may explain the fact that—compared with Msalala—Kilolo shows a larger number of cases directed towards the regional referral hospital. In the case of Msalala district, the closest district referral hospital is located in the neighbouring Kahama urban district.

Figure 3 shows the different referral networks in the two districts, with nodes size proportional to betweenness score. Edges represent the existence of at least one referral between two facilities. In both districts, the networks related to treatment of childhood illnesses have slightly higher density. The structural patterns observed in the networks of childcare referrals do not differ compared with those for treatment of NCDs. In Kilolo, two facilities polarize most incoming referrals: the regional referral hospital (Iringa Town) and the Ililula district designated hospital. Concerning referrals for treatment of childhood illnesses, health centres appear moderately connected. For referrals related to treatment of NCDs, health centres are marginal in the network. In Msalala, although the Kahama hospital is very central in both networks, the referrals are more uniformly distributed. Health centres are well connected in the referrals network, receiving referrals from dispensaries and sending referrals to Kahama hospital. Interestingly, the Lunguya health centre has a relevant gatekeeping role for childcare which is not observed for treatment of NCDs. The opposite is true for Chela health centre, which attracts several patient referrals in the domain of NCDs but much less among sick children. These changes are consistent with a recognized proficiency at Lunguya and Chela in treating sick children and patients affected by NCDs, respectively.

Table 5 reports ranking of in-degree and betweenness score for treatment of childhood illnesses and NCDs. In Kilolo, the regional referral hospital in Iringa Town attracts most of referrals, followed by a district designated hospital (Ililula, FBO) and a health centre (Kidabaga). The health facility that emerges with highest betweenness score is the Ililula district designated hospital. Whilst from these figures, we cannot infer the reasons for the observed patterns of referrals, we can state that the referral flows show a marginal role of health centres in Kilolo. Most prominently, the regional referral hospital in Iringa town plays a key role. The large number of referrals towards hospitals is likely to contribute to an increased burden of hospital wards at regional and district hospitals.

**Table 5:** Facilities with highest In-degree and betweenness scores for referrals in Kilolo and Msalala districts

*Treatment of childhood illnesses*				*Treatment of NCDs*			
*In-degree*	*Value*	*Betweenness*	*Value*	*In-degree*	*Value*	*Betweenness*	*Value*
Kilolo DC							
Regional Hospital	18	Ililula hospital	6	Regional hospital	16	Ililula hospital	13
Ililula Hospital	7	Kibadaga health centre	5	Ililula hospital	14		
Kidabaga health centre	2			Kidabaga health centre	2		
Msalala DC							
Kahama hospital	17	Lunguya health centre	4	Kahama hospital	11	Chela health centre	4
Chela health centre	5	Segese dispensary	2	Chela health centre	4		
Lunguya health centre	4	Bugarama health centre	2	Lunguya health centre	2		

In Msalala, the Kahama district hospital has the highest number of incoming referrals, followed by two health centres (Lunguya and Chela). Health centres also have high betweenness score. This indicates that the network position of health centres in Msalala is consistent with the role of gatekeepers for tertiary specialized care provided in hospitals. As suggested above, the large number of referrals reaching the Kahama hospital should not surprise, provided that it is the closest district hospital.

### Analysis of referral network determinants

All the estimated ERGMs show no signs of degeneracy and perform well in all standard goodness-of-fit tests. Detailed diagnostics are included in Supplementary Appendix. Table 6 reports results for models fitted on the networks of referrals related to treatment of childhood illnesses in the two districts. In Kilolo, distance among facilities decreases the conditional likelihood of tie formation, for both model specifications. Consistently with the networks represented in figures above, hospitals appear to be more likely than health centres to have incoming referrals. The number of beds at facility level does not appear to influence network formation. On the other hand, the coefficient for the number of rooms in model 2 suggests that—conditionally on other characteristics—the size of the facility increases slightly the log-odds of a tie. Furthermore, our models do not detect any influence of availability of transportation means on network formation. The number of facility deliveries is also not associated to probability of tie formation in our model.

**Table 6:** ERGMs for referral networks related to treatment of childhood illnesses

	Kilolo DC		Msalala MC	
	Model 1	Model 2	Model 1	Model 2
Edges	−11.62* (4.69)	−10.70* (4.16)	−3.78 (2.66)	−24.06*** (7.20)
Isolates	−0.06 (0.61)	−0.11 (0.61)	−0.83 (0.77)	−0.67 (0.77)
Geometrically weighted in-degree distribution (GWIDEG)	4.61 (3.23)	3.82 (2.67)	−0.58 (1.61)	10.54** (3.89)
Edge covariate: road distance (KM)	−1.78** (0.64)	−1.86** (0.65)	−3.74*** (0.72)	−3.57*** (0.72)
Incoming ties, node factor: health centre	6.52** (2.22)	5.70** (1.93)	4.05* (1.90)	11.86** (2.78)
Incoming ties, node factor: hospital	10.46** (3.66)	9.62** (3.41)	−2.02 (9.81)	19.78*** (3.64)
Incoming ties, node covariate: delivery beds	−0.24 (0.56)	−0.39 (0.54)	1.19** (0.45)	2.21* (0.92)
Outgoing ties, node covariate: delivery beds	0.27 (0.28)	0.19 (0.30)	0.39 (0.64)	0.68 (0.66)
Incoming ties, node covariate: patient beds	−0.01 (0.02)	−0.01 (0.01)	0.04 (0.04)	0.10 (0.07)
Outgoing ties, node covariate: patient beds	−0.04 (0.02)	−0.04 (0.02)	−0.05 (0.04)	
Incoming ties, node covariate: no. of rooms			−0.15 (0.29)	−2.90*** (1.03)
Outgoing ties, node covariate: no. of rooms			−0.20 (0.25)	−0.35 (0.20)
Combined node covariate: no. of rooms	0.30 (0.16)	0.32* (0.16)		
Node covariate: no. of motorcycles	−0.31 (0.56)	−0.41 (0.56)	0.07 (0.62)	−0.43 (0.66)
Node covariate: number of ambulances	0.13 (0.66)	0.12 (0.63)	0.34 (0.73)	−0.01 (0.80)
Node covariate: log of catchment population	−0.14 (0.36)	−0.25 (0.38)	−0.01 (0.27)	−0.07 (0.26)
Incoming ties, log number of facility deliveries		0.22 (0.27)		4.46*** (1.38)
GWIDEG decay parameter	0.7	0.7	0.5	1.2
Akaike Information Criterion	186.67	188.03	145.18	132.97
Bayesian Information Criterion	265.56	272.56	2217.70	205.50
Log-likelihood	−79.33	−79.01	−57.59	−51.48

*Notes:* Coefficients represent contributions to log-odds. Standard errors are in parentheses.

*** *P* < 0.001, **P < 0.01, *P < 0.05.

In Shinyanga, distance between facilities is associated to lower odds of referral. Conditionally on all variables included in the models, incoming referrals are more likely in facilities with larger labour wards. The inclusion of the number of facility deliveries in model 2 changes the estimate associated with the type of facility. In model 1, the coefficient associated to health centres is large and positive whilst hospitals show no significant association. In model 2, consistently with the figures, hospitals are more likely than health centres to have incoming referrals. Compared with model 1, model 2 also shows a negative coefficient associated to number of rooms for incoming ties. This suggests that smaller facilities are more likely to attract referrals. Finally, the coefficient associated to facility deliveries is fairly large and significant.

To put these results into perspective, let us consider a referral originating from a dispensary with 4 rooms and 1 delivery bed and 19 deliveries in the previous 3 months (our median facility). Moreover, let us consider a potential referral to a health centre 5 km away with 4 delivery beds, 40 rooms and 90 deliveries in the previous 3 months. For Kilolo, our model suggests that the health centre would experience a 35% increase in the probability of incoming referral expanding its infrastructure from 40 to 45 rooms. In Msalala, for an analogous pair of facilities, the health centre would experience a 16% increase in the probability of an incoming referral with 150 instead of 100 deliveries in the previous 3 months. It is worth noting that the intensity of the effect depends on the initial conditions (i.e. change statistics) representing the edge and the characteristics of the pair of facilities evaluated.

Table 7 reports the results of ERGMs fitted on the networks of referrals related to treatment of NCDs. Overall, the ERGMs results are consistent with the geographical distribution of health facilities and patient referrals for treatment of NCDs represented in Figures 1 and 2. Similarly to referrals for treatment of childhood illnesses, both districts are characterized by higher tendency of referrals between facilities that are geographically close to each other. Furthermore, hospitals are more likely to receive referrals compared with health centres. For Kilolo, outgoing referrals seem to be slightly less likely for facilities with more beds and smaller catchment areas. The number of rooms is associated with higher likelihood of a tie, although we could not separate effects for senders and receivers.

**Table 7:** ERGMs for networks of referrals related to treatment of NCDs

	Kilolo DC	Msalala MC
Edges	34.97*** (9.47)	−0.68 (1.89)
Isolates	−1.69* (0.76)	0.07 (0.60)
Geometrically weighted in-degree distribution (GWIDEG)	9.83 (4.19)	−3.19*** (0.85)
Edge covariate: geographic distance (KM)	−2.33*** (0.76)	−1.74* (0.74)
Incoming ties, node factor: health centre		2.31** (0.85)
Incoming ties, node factor: hospital		6.07*** (0.31)
Combined node covariate: health centre	3.84** (1.40)	
Combined node covariate: hospital	15.71*** (4.04)	
Incoming ties, node covariate: patient beds	−0.07*** (0.02)	0.01 (0.02)
Outgoing ties, node covariate: patient beds	−0.25*** (0.06)	
Incoming ties, node covariate: no. of rooms		−0.27 (0.18)
Outgoing ties, node covariate: no. of rooms		−0.13 (0.14)
Combined node covariate: no. of rooms	0.65** (0.22)	
Incoming ties, node covariate: no. of motorcycles		0.96* (0.40)
Outgoing ties, node covariate: no. of motorcycles	0.21 (0.69)	−0.04 (0.61)
Node covariate: no. of ambulances	1.02 (0.94)	0.07 (0.71)
Outgoing ties, node covariate: log of catchment population	1.54** (0.60)	−0.04 (0.22)
GWIDEG decay parameter	2.25	0.9
Akaike Information Criterion	141.24	133.58
Bayesian Information Criterion	208.87	196.44
Log-likelihood	−58.62	−53.79

*Notes:* Coefficients represent contributions to log-odds. Standard errors are in parentheses.

*** *P* < 0.001, **P < 0.01, *P < 0.05.

## Discussion

The results show two contrasting situations. In Kilolo, the majority of patient referrals are directed towards the regional and district referral hospitals. Health centres play a minor role, with few incoming referrals and limited gatekeeping activity as measured by the betweenness indicator. The latter deviations from standard district referral flows can arise for multiple reasons, including: geographical closeness, better quality of care, community preferences or pre-existing ties between individual providers. The geographic distribution of facilities suggests that the disproportionate share of referrals directed towards the regional referral hospital in Iringa Town might be related to geographical proximity. The analysis of the determinants of patient referrals network supports this interpretation. Notably, ERGMs results also reveal that the only facility characteristic, among those investigated, associated with the emergence of childcare referrals is the number of rooms. For patient referrals related to treatment of NCDs, a higher number of inpatient beds are associated to lower odds of referring patients whilst a higher number of rooms are generally associated with increased probability of a tie. Our analysis suggests that the local health system in Kilolo relies heavily on tertiary hospital care and not much on secondary care provided by hospitals. Whilst this may be clinically appropriate, it could be expensive and cause financial hardship for the households required to move to the referral facility.

In Msalala, we observe a concentration of referrals directed towards the closer district hospital in Kahama district, as Msalala does not have a tertiary-level facility. However, the referral networks indicate an important role for health centres, which attract referrals from dispensaries acting effectively as gatekeepers. This is especially true for the network of referrals related to treatment of childhood illnesses. Compared with Kilolo, the travel distance from many dispensaries to the closest hospitals is larger. Conditionally on the other relevant factors, this likely contributes to the higher rate of referrals directed to health centres, compared with Kilolo. The ERGM analysis of referrals for treatment of childhood illnesses in Msalala suggests smaller facilities with higher number of deliveries seem to be more likely to receive a patient referral. A possible interpretation for this result is that of a virtuous cycle; specialization may be recognized as proxy of quality and thus trigger more incoming referrals to closer and smaller facilities. With regards to patient referrals related to NCDs, the number of motorcycles available in the facilities seems to be weakly associated to the likelihood of a patient referral between two facilities. This supports the idea that transportation infrastructure facilitates patient referrals. We are, however, careful in over interpreting the result as the absence of a similar association for Kilolo is likely related to different geographic characteristics. The mountainous territory in Kilolo is impervious compared with the lowland in Msalala.

Our analysis points to the importance of adequate investments in infrastructure for intermediate secondary-level referral facilities (Mwangome *et al.*, 2017). For instance, there is compelling evidence the rural and mountainous Kilolo district saw few funds allocated to construction and maintenance of secondary and tertiary facilities, to means of transportation (Kigume and Maluka, 2018b) and staffing (Maluka *et al.*, 2020). Consequently, an FBO-managed hospital is designated as district referral hospital, which may discourage attendance of poor households imposing user fees, contrary to public facilities (Saksena *et al.*, 2010). Furthermore, many patients in need of referral are directly sent to the regional referral in Iringa Town, whilst health centres do not appear to receive referrals from nearby dispensaries. These well-known disruptions in the intended referral flows may further discourage access to care due to high travel expenses (Saksena *et al.*, 2010) and specific cultural policies, e.g. spouse accompany (Maluka *et al.*, 2020). Lastly, health facilities located close to district borders—or in districts that do not have a tertiary-level facility—naturally refer patients to other facilities located in neighbouring district not included in our analysis.

Finally, households required to travel to a district or regional hospital face a high financial and organizational burden. Therefore, the interpretation of referrals from dispensaries to hospitals being driven by explicit patient request appears unlikely. Additionally, the current regulation of public healthcare provision exempts childcare and NCDs care from payments across all public facilities and does not attach specific financial incentives to patient referrals. Accordingly, the hypothesis of distorted incentives at the level of dispensaries and towards referring patients to hospitals rather than health centres seems likewise implausible.

Nevertheless, the study has several limitations. First of all, we analysed primary data from a survey conducted on public facilities only. Private healthcare providers are embedded in health systems and treat a substantial share of patients across Tanzania, including Kilolo and Msalala districts. A better representation of patient referrals should include all providers. Specifically, whilst we captured some referrals towards private providers, it is important to also report on referrals from private providers towards other facilities. Secondly, data were collected from the sending health facilities, without details about the clinical and organizational appropriateness, success and outcomes of patient referrals. A complete assessment of the effectiveness of referral systems cannot be conducted without properly considering its relevance and the associated patient outcomes. Regarding the last remarks, constant improvements in health information systems and routine data collection across LMICs provide interesting opportunities for future research (Harries *et al.*, 2013; Wagenaar *et al.*, 2016). Third, the ERGMs employed to study determinants can potentially reveal mechanisms that are hard to identify with a qualitative approach due to intrinsic limits on the number of factors that a researcher can consider (Kim *et al.*, 2016). However, standard ERGMs present many limitations (Desmarais and Cranmer, 2012), including risk of degeneracy associated to MCMC estimation and inability to deal with missing data. For example, the latter limitation restricted our analysis on a subset of facility characteristics that were available for all network nodes. Fourth, the representation of the estimated effects could be improved by the use of graphs showing marginal probabilities across a range of values of the covariates of interest. This visual tool was out of the explorative scope of this analysis but could be included in network analyses focused on the effect of specific characteristics. To this extent, it is important to stress that marginal probability depends upon the change statistics associated to the type of edge and the pair of nodes of interest. In general, the quantification of network-wide average effects is only meaningful for intrinsic network characteristics, such as edge propensity. Finally, our study has limited external validity: we only analysed two district health systems that do not represent the full spectrum of district health systems in Tanzania.

## Conclusion

This study showed the potential of using network analysis to assess patient referrals. The results of the study highlight the need for Tanzanian authorities to tackle the central issue of patient referrals from dispensaries. Viable policies might include strengthening physical and transport infrastructures at health centres, improving staffing, training and procedures in secondary-level facilities and possibly setting up a new system of financial and non-financial incentives rewarding successful patient referrals. Improved referral to secondary-level facilities could avoid unnecessary referrals to hospitals, reducing the travel-related burden for households and expensive hospital care.

## Supplementary data

Supplementary data are available at *Health Policy and Planning* online.

## Funding

This work is an output from the project ‘Health systems governance for an inclusive and sustainable social health protection in Ghana and Tanzania’ funded by the Swiss Programme for Research on Global Issues for Development [grant number 160373], jointly financed by the Swiss National Science Foundation and the Swiss Agency for Development and Cooperation. The project involved a consortium of five partners: Swiss Tropical and Public Health Institute, ETH Zurich, University of Applied Sciences and Arts of Southern Switzerland (SUPSI), Ifakara Health Institute Tanzania and University of Ghana.

## Supplementary Material

czaa138_Supplementary_Data
