# Supplementary material for: A network analysis of patient referrals in two district health systems in Tanzania
Source: Health Policy Plan. 2020 Dec 24;36(2):162–75. doi: 10.1093/heapol/czaa138 (PMC7996649; doi:10.1093/heapol/czaa138)
Supplement: czaa138_Supplementary_Data [file czaa138_supplementary_data.zip › 20200618_figure2.docx]

Figure 2: Maps of patient referrals for Msalala district, Shinyanga Region.


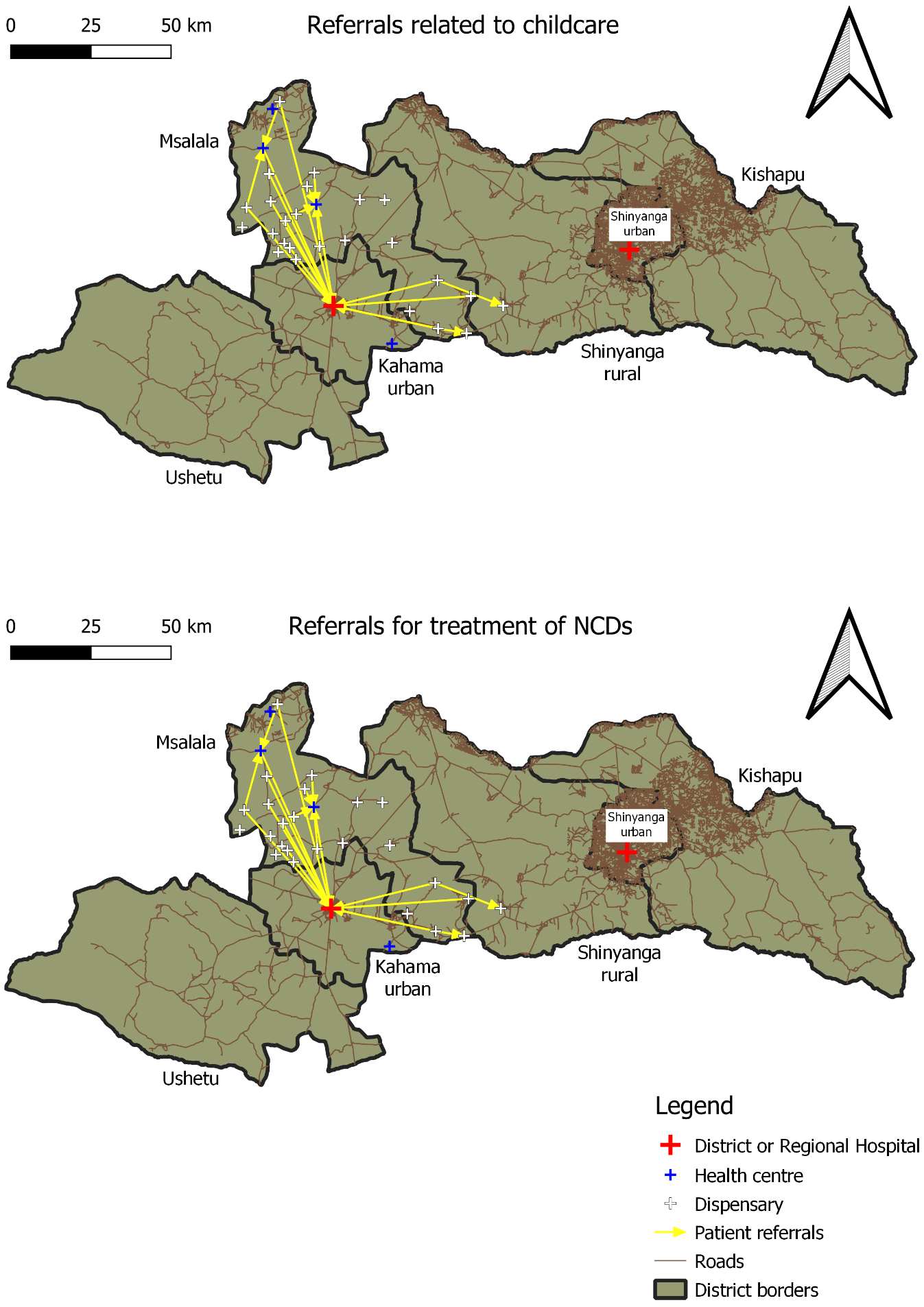


*Note: District names reported outside of district borders.*
