# Supplementary material for: A network analysis of patient referrals in two district health systems in Tanzania
Source: Health Policy Plan. 2020 Dec 24;36(2):162–75. doi: 10.1093/heapol/czaa138 (PMC7996649; doi:10.1093/heapol/czaa138)
Supplement: czaa138_Supplementary_Data [file czaa138_supplementary_data.zip › 20200618_figure3.docx]

Figure 3: Sociograms for referral networks in Kilolo DC and Msalala DC with nodes size proportional to betweenness score.

| Kilolo DC | Msalala DC |
| --- | --- |
| 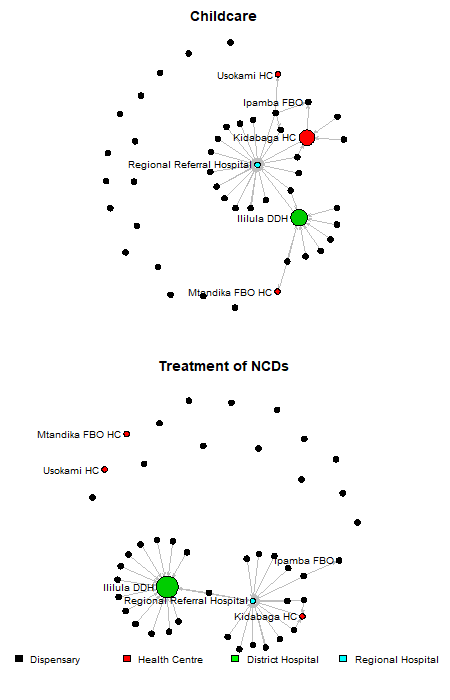 | 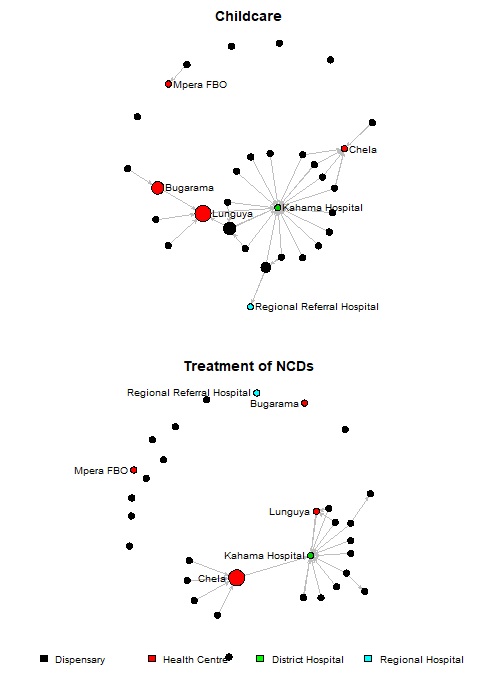 |

*Note: Dots represent facilities, arrows indicate referrals. Names of the facilities are reported for health centres and hospitals only.*
