# Supplementary material for: A network analysis of patient referrals in two district health systems in Tanzania
Source: Health Policy Plan. 2020 Dec 24;36(2):162–75. doi: 10.1093/heapol/czaa138 (PMC7996649; doi:10.1093/heapol/czaa138)
Supplement: czaa138_Supplementary_Data [file czaa138_supplementary_data.zip › 20200904_appendix_anonymous.pdf]

A network analysis of patient referrals in two district health systems in Tanzania

Version 04.09.2020

A Online appendix

A.1 Health facility types and roles in the Tanzanian public sector

	Dispensary	Health centre	District referral hospital	Regional referral hospital
Number of public facilities across the country	~5200	~600	73<br>41 non-public designated	27
Level	Primary	Secondary	Tertiary	Tertiary
Refers patients to	Health centre in catchment area	District or regional referral hospitals	Regional referral or super specialized hospitals	National or super specialized hospitals
Suggested wards	1	3	7	9+
Suggested inpatient beds	-	20-30	100-175	176-450
Suggested number of staff	10-20	40-60	200-312	468-680
Most qualified cadres		Medical doctor	Specialized medical doctor (surgery)	Specialized medical doctors
Services covered	. OPD / Emergency<br>. RCH clinic<br>. Delivery<br>. Community services<br>. Laboratory	. OPD / Emergency<br>. Eye care services<br>. Primary oral health<br>. RCH clinic<br>. VCT service<br>. CTC service<br>. Home-based care<br>. Community services<br>. Laboratory<br>. Mortuary<br>. Pharmacy	. General medicine<br>. Casualty and emergency<br>. Surgery<br>. Internal medicine<br>. Obstetrics<br>. Gynaecology<br>. Physiotherapy<br>. RCH clinic<br>. Geriatric services<br>. Nutrition<br>. Oral health<br>. Eye care services<br>. Mental health<br>. Laboratory<br>. Radiology<br>. Pharmacy<br>. Nutrition	. General medicine<br>. Casualty and emergency<br>. Surgery<br>. Internal medicine<br>. Orthopaedic clinic<br>. Obstetrics<br>. Gynaecology<br>. Physiotherapy<br>. Paediatric<br>. Dental clinic<br>. CTC clinic<br>. Ear, nose & throat clinic<br>. RCH clinic<br>. Geriatric services<br>. Eye care services<br>. Psychiatric clinic<br>. TB ward<br>. Intensive care unit<br>. Sterilization<br>. Laboratory<br>. Mortuary<br>. X-Ray<br>. Pharmacy<br>. Environmental health, food safety and sanitation

Source: Ministry of Health and Social Welfare. 2014. "Staffing Levels for Ministry of Health and Social Welfare Departments, Health Service Facilities, Health Training Institutions and Agencies 2014-2019." Dar es Salaam, Tanzania: United Republic of Tanzania.

The structure of public health service delivery in Tanzania is highly decentralized. The box summarizes facility types and roles as reflected by guidelines issued by the Tanzanian Ministry of Health. This pyramidal structure builds on a large number of dispensaries, facilities that cover catchment areas of about 6'000 to 10'000 people. According to official government guidelines, the most qualified cadres assigned to dispensaries are clinical officers and nurses. Staff is required to perform basic primary care procedures. At the secondary level, health centres cover about 50'000 people and are the first referral level from primary level facilities in their catchment areas, except for complicated

cases. Health centres are better equipped in terms of infrastructure and technology. Furthermore, health centres employ trained personnel including medical doctors, assistant medical officers, radiographer technologists, health laboratory and pharmaceutical technologists and assistant dental officers. The range of service providers is sensibly larger compared to dispensaries, as reflected by the box below. At the top of the structure, district and regional referral hospitals cover the full range of health services needed by the population, except for highly specialized care which is delegated to national and specialized referral hospitals. District and regional hospitals are the last referral point at the district and respectively regional level. They should receive referrals from secondary level facilities or – in rare complicated cases – directly from dispensaries.

A.2 Potential referral conditions addressed by the survey.

**Area**	**Description**
Childcare	
Emergency conditions	Issues related to triage and emergency conditions.
Diagnostic approaches	Specific issues related to the diagnosis of the condition affecting children.
Cough or difficulty breathing	Issues related to treatment of cough or difficulty breathing.
Diarrhoea	Issues related to treatment of diarrhoea.
Fever	Issues related to treatment of feverish conditions.
Severe acute malnutrition	Issues related to treatment of severe acute malnutrition.
Children with HIV/AIDS	Issues related to treatment of HIV/AIDS.
Surgical problems	Issues related to problems with surgical procedures.
Supportive care	Issues related oxygen therapy, choice of intravenous fluids and treatment of hypoglycaemia.
Non-communicable diseases	
Diabetes	Issues related to diabetes care.
Cancer	Issues related to treatment of any type of cancer.
Cardiovascular diseases	Issues related to treatment of cardiovascular diseases.
Chronic respiratory diseases	Issues related to treatment of any chronic respiratory disease.
Mental health conditions	Issues related to treatment of any mental health condition.

Note: the patient referral system was assessed in relation to clinical matters; referral related to pharmacology; advice related to clinical matter and advice related to pharmacology.

A.3 Survey questionnaire

Below we reproduce the paper version of the full questionnaire used during fieldwork between April and July 2018. The mention "*See list*" refers to the list of health facilities within the district/region. The data collection was performed using the ODK tablet version of the questionnaire.

Research title: Mapping patterns of district level health system governance network structure. Implications for social health protection, access to healthcare and health systems performance in Ghana and Tanzania

Section 1: Characteristics of the health facility

Q1	Name of the health facility	*See list*
Q2	Location (Village, Ward, District, Region)	
Q3	GPS coordinates	Latitude
		Longitude
Q4	Type	Dispensary □<br>Health center □<br>District hospital □<br>Regional hospital □
Q5	Total staff assigned	
Q6	Number of nurses	
Q7	Number of MDs	
Q8	Number of beds OPD	
Q9	Number of beds IPD	
Q10	Number of beds RCH clinic	
Q11	Does the facility store vaccines	Yes □ No □
Q12	Does the facility store other medicines?	Yes □ No □
Q13	Number of OPD visits last 3 months	
Q14	Number of IPD visits last 3 months	
Q15	Number of RCH clinic visits last 3 months	
Q16	Is there a private health facility close by (same village or ward)	No □<br>Yes □
Q16.1	Distance from private health facility	Up to 2 km □<br>From 3 to 10 km □<br>From 11 to 30 km □<br>More than 30 km □
Q17	Private pharmacy close by (same village or ward)	No □<br>Yes □
Q17.1	Distance from private pharmacy	Up to 2 km □<br>From 3 to 10 km □<br>From 11 to 30 km □<br>More than 30 km □
Q18	Is there a cascade supervision from higher level facility	No □<br>Yes □
Q18.1	What is the name of reference facility for cascade supervision	*See list*

Section 2: Relational data: child care services

Characteristics of the respondent

Q19	First Name and Last Name	
Q20	Date of birth	
Q21	Place of birth (Village, Ward, District, Region)	

Q22	Role in the facility / office	HF incharge □<br>MD □<br>Clinician □<br>Nurse □<br>Midwife □<br>Clinical assistant □<br>Other medical staff □<br>Administrative staff □<br>Department head □<br>Specify role: ________
Q23	For hospitals and health centers, in which department are you working?	
Q24	How long have you worked at this health facility?	Years _______ Months________
Q25	Last highest educational title	
Q26	Place where last education was completed (District, Region)	
Q27	Number of nurses in the department	
Q28	Number of MDs in the department	
Q29	Number of beds OPD in the department	
Q30	Number of beds IPD in the department	
Q31	Number of beds RCH clinic in the department	
Q32	Is there any referral or advice given for clinical or pharmacological matters related to "**Emergency conditions**" of a sick child in the last 3 months?<br><br>If yes, for the last event:	No □<br>Yes □
Q32.1	What was the type of interaction	Referral, clinical matter □<br>Referral, pharmacology □<br>Advice, clinical matter □<br>Advice, pharmacology □<br>Other (specify): _____________
Q32.2	Which health facility / office? Select from the list.	*See list*
Q32.3	First and Last name of contact person	
Q32.4	Role of contact person in the facility / office	HF incharge □<br>MD □<br>Clinician □<br>Nurse □<br>Midwife □<br>Clinical assistant □<br>Other medical staff □<br>Administrative staff □<br>DMO office □<br>Specify role: ________
Q32.5	Relationship with the contact person	Only work-related □<br>Friend □<br>Family □<br>Former colleague □<br>Schoolmate □

Q32.6	How did you get in contact with the contact person	In person □<br>Phonecall □<br>Text message □<br>Email □<br>Regular mail □<br>Other (specify) __________<br>______________________________
Q32.7	How do you evaluate the success of the specific referral, on a scale from 1 to 4 where 1 is failure and 4 is complete success?	Failure Complete success<br>□ 1 □ 2 □ 3 □ 4
Q33	Referral or advice for clinical or pharmacological matters related to "**Diagnostic approaches to sick child**" of a sick child in the last 3 months?<br><br>If yes, for the last event:	No □<br>Yes □
Q33.1	Type of interaction	Referral, clinical matter □<br>Referral, pharmacology □<br>Advice, clinical matter □<br>Advice, pharmacology □<br>Other (specify): ______________
Q33.2	Which health facility / office? Select from the list.	*See list*
Q33.3	First and Last name of contact person	
Q.33.4	Role of contact person in the facility / office	HF incharge □<br>MD □<br>Clinician □<br>Nurse □<br>Midwife □<br>Clinical assistant □<br>Other medical staff □<br>Administrative staff □<br>DMO office □<br>Specify role: _________
Q33.5	Relationship with the contact person	Only work-related □<br>Friend □<br>Family □<br>Former colleague □<br>Schoolmate □
Q33.6	How did you get in contact with the contact person	In person □<br>Phonecall □<br>Text message □<br>Email □<br>Regular mail □<br>Other (specify) __________<br>______________________________

Q33.7	How do you evaluate the success of the specific referral, on a scale from 1 to 4 where 1 is failure and 4 is complete success?	Failure Complete success<br>□ 1 □ 2 □ 3 □ 4
Q34	Referral or advice for clinical or pharmacological matters related to "**Cough or difficulty in breathing**" of a sick child in the last 3 months?<br><br>If yes, for the last event:	No □<br>Yes □
Q34.1	Type of interaction	Referral, clinical matter □<br>Referral, pharmacology □<br>Advice, clinical matter □<br>Advice, pharmacology □<br>Other (specify): ______________
Q34.2	Which health facility / office? Select from the list.	*See list*
Q34.3	First and Last name of contact person	
Q34.4	Role of contact person in the facility / office	HF incharge □<br>MD □<br>Clinician □<br>Nurse □<br>Midwife □<br>Clinical assistant □<br>Other medical staff □<br>Administrative staff □<br>DMO office □<br>Specify role: _________
Q34.5	Relationship with the contact person	Only work-related □<br>Friend □<br>Family □<br>Former colleague □<br>Schoolmate □
Q34.6	How did you get in contact with the contact person	In person □<br>Phonecall □<br>Text message □<br>Email □<br>Regular mail □<br>Other (specify) __________<br>______________________________
Q34.7	How do you evaluate the success of the specific referral, on a scale from 1 to 4 where 1 is failure and 4 is complete success?	Failure Complete success<br>□ 1 □ 2 □ 3 □ 4
Q35	Referral or advice for clinical or pharmacological matters related to "**Diarrhoea**" of a sick child in the last 3 months?<br><br>If yes, for the last event:	No □<br>Yes □

Q35.1	Type of interaction	Referral, clinical matter ☐<br>Referral, pharmacology ☐<br>Advice, clinical matter ☐<br>Advice, pharmacology ☐<br>Other (specify): _____________
Q35.2	Which health facility / office? Select from the list.	*See list*
Q35.3	First and Last name of contact person	
Q35.4	Role of contact person in the facility / office	HF incharge ☐<br>MD ☐<br>Clinician ☐<br>Nurse ☐<br>Midwife ☐<br>Clinical assistant ☐<br>Other medical staff ☐<br>Administrative staff ☐<br>DMO office ☐<br>Specify role: ________
Q35.5	Relationship with the contact person	Only work-related ☐<br>Friend ☐<br>Family ☐<br>Former colleague ☐<br>Schoolmate ☐
Q35.6	How did you get in contact with the contact person	In person ☐<br>Phonecall ☐<br>Text message ☐<br>Email ☐<br>Regular mail ☐<br>Other (specify) _________<br>____________________________
Q35.7	How do you evaluate the success of the specific referral, on a scale from 1 to 4 where 1 is failure and 4 is complete success?	Failure Complete success<br>☐ 1 ☐ 2 ☐ 3 ☐ 4
Q36	Referral or advice for clinical or pharmacological matters related to "**Fever**" of a sick child in the last 3 months?<br><br>If yes, for the last event:	No ☐<br>Yes ☐
Q36.1	Type of interaction	Referral, clinical matter ☐<br>Referral, pharmacology ☐<br>Advice, clinical matter ☐<br>Advice, pharmacology ☐<br>Other (specify): _____________
Q36.2	Which health facility / office? Select from the list.	*See list*
Q36.3	First and Last name of contact person	

Q36.4	Role of contact person in the facility / office	HF incharge □<br>MD □<br>Clinician □<br>Nurse □<br>Midwife □<br>Clinical assistant □<br>Other medical staff □<br>Administrative staff □<br>DMO office □<br>Specify role: _________
Q36.5	Relationship with the contact person	Only work-related □<br>Friend □<br>Family □<br>Former colleague □<br>Schoolmate □
Q36.6	How did you get in contact with the contact person	In person □<br>Phonecall □<br>Text message □<br>Email □<br>Regular mail □<br>Other (specify) __________<br>______________________________
Q36.7	How do you evaluate the success of the specific referral, on a scale from 1 to 4 where 1 is failure and 4 is complete success?	Failure ... Complete success<br>□ 1 □ 2 □ 3 □ 4
Q37	Referral or advice for clinical or pharmacological matters related to "**Severe acute malnutrition**" of a sick child in the last 3 months?<br><br>If yes, for the last event:	No □<br>Yes □
Q37.1	Type of interaction	Referral, clinical matter □<br>Referral, pharmacology □<br>Advice, clinical matter □<br>Advice, pharmacology □<br>Other (specify): ______________
Q37.2	Which health facility / office? Select from the list.	*See list*
Q37.3	First and Last name of contact person	
Q37.4	Role of contact person in the facility / office	HF incharge □<br>MD □<br>Clinician □<br>Nurse □<br>Midwife □<br>Clinical assistant □<br>Other medical staff □<br>Administrative staff □<br>DMO office □<br>Specify role: _________

Q37.5	Relationship with the contact person	Only work-related ☐<br>Friend ☐<br>Family ☐<br>Former colleague ☐<br>Schoolmate ☐
Q37.6	How did you get in contact with the contact person	In person ☐<br>Phonecall ☐<br>Text message ☐<br>Email ☐<br>Regular mail ☐<br>Other (specify) __________<br>______________________________
Q37.7	How do you evaluate the success of the specific referral, on a scale from 1 to 4 where 1 is failure and 4 is complete success?	Failure Complete success<br>☐ ☐ ☐ ☐<br>1 2 3 4
Q38	Referral or advice for clinical or pharmacological matters related to "**Children with HIV/AIDS**" in the last 3 months?<br><br>If yes, for the last event:	No ☐<br>Yes ☐
Q38.1	Type of interaction	Referral, clinical matter ☐<br>Referral, pharmacology ☐<br>Advice, clinical matter ☐<br>Advice, pharmacology ☐<br>Other (specify): ______________
Q38.2	Which health facility / office? Select from the list.	*See list*
Q38.3	First and Last name of contact person	
Q38.4	Role of contact person in the facility / office	HF incharge ☐<br>MD ☐<br>Clinician ☐<br>Nurse ☐<br>Midwife ☐<br>Clinical assistant ☐<br>Other medical staff ☐<br>Administrative staff ☐<br>DMO office ☐<br>Specify role: _________
Q38.5	Relationship with the contact person	Only work-related ☐<br>Friend ☐<br>Family ☐<br>Former colleague ☐<br>Schoolmate ☐
Q38.6	How did you get in contact with the contact person	In person ☐<br>Phonecall ☐<br>Text message ☐<br>Email ☐<br>Regular mail ☐<br>Other (specify) __________

Q38.7	How do you evaluate the success of the specific referral, on a scale from 1 to 4 where 1 is failure and 4 is complete success?	Failure Complete success<br>□ 1 □ 2 □ 3 □ 4
Q39	Referral or advice for clinical or pharmacological matters related to “**Surgical problems**” of a sick child in the last 3 months?<br><br>If yes, for the last event:	No □<br>Yes □
Q39.1	Type of interaction	Referral, clinical matter □<br>Referral, pharmacology □<br>Advice, clinical matter □<br>Advice, pharmacology □<br>Other (specify): ______________
Q39.2	Which health facility / office? Select from the list.	*See list*
Q39.3	First and Last name of contact person	
Q39.4	Role of contact person in the facility / office	HF incharge □<br>MD □<br>Clinician □<br>Nurse □<br>Midwife □<br>Clinical assistant □<br>Other medical staff □<br>Administrative staff □<br>DMO office □<br>Specify role: _________
Q39.5	Relationship with the contact person	Only work-related □<br>Friend □<br>Family □<br>Former colleague □<br>Schoolmate □
Q39.6	How did you get in contact with the contact person	In person □<br>Phonecall □<br>Text message □<br>Email □<br>Regular mail □<br>Other (specify) __________<br>______________________________
Q39.7	How do you evaluate the success of the specific referral, on a scale from 1 to 4 where 1 is failure and 4 is complete success?	Failure Complete success<br>□ 1 □ 2 □ 3 □ 4
Q40	Referral or advice for clinical or pharmacological matters related to “**Supportive care**” (e.g. nutritional management, breastfeeding, pain control) of a sick child in the last 3 months?<br><br>If yes, for the last event:	No □<br>Yes □

Q40.1	Type of interaction	Referral, clinical matter ☐<br>Referral, pharmacology ☐<br>Advice, clinical matter ☐<br>Advice, pharmacology ☐<br>Other (specify): ______________
Q40.2	Which health facility / office? Select from the list.	*See list*
Q40.3	First and Last name of contact person	
Q40.4	Role of contact person in the facility / office	HF incharge ☐<br>MD ☐<br>Clinician ☐<br>Nurse ☐<br>Midwife ☐<br>Clinical assistant ☐<br>Other medical staff ☐<br>Administrative staff ☐<br>DMO office ☐<br>Specify role: _________
Q40.5	Relationship with the contact person	Only work-related ☐<br>Friend ☐<br>Family ☐<br>Former colleague ☐<br>Schoolmate ☐
Q40.6	How did you get in contact with the contact person	In person ☐<br>Phonecall ☐<br>Text message ☐<br>Email ☐<br>Regular mail ☐<br>Other (specify) __________<br>______________________________
Q40.7	How do you evaluate the success of the specific referral, on a scale from 1 to 4 where 1 is failure and 4 is complete success?	Failure — Complete success<br>☐ 1 ☐ 2 ☐ 3 ☐ 4

Section 3: Relational data: care for chronic conditions

Characteristics of the respondent

Q41	First Name and Last Name	
Q42	Date of birth	
Q43	Place of birth (Village, Ward, District, Region)	
Q44	Role in the facility / office	HF incharge ☐<br>MD ☐<br>Clinician ☐<br>Nurse ☐<br>Midwife ☐<br>Clinical assistant ☐<br>Other medical staff ☐<br>Administrative staff ☐<br>Department head ☐<br>Specify role: ________
Q45	For hospitals and health centers, in which department are you working?	
Q46	How long have you worked at this health facility?	Years _______ Months________
Q47	Last highest educational title	
Q48	Place where last education was completed (District, Region)	
Q49	Number of nurses in the department	
Q50	Number of MDs in the department	
Q51	Number of beds OPD in the department	
Q52	Referral or advice for clinical or pharmacological matters related to the diagnosis or treatment of a patient affected by “**Diabetes**” in the last 3 months?<br><br>If yes, for the last event:	No ☐<br>Yes, diagnosis ☐<br>Yes, treatment ☐
Q52.1	Type of interaction	Referral, clinical matter ☐<br>Referral, pharmacology ☐<br>Advice, clinical matter ☐<br>Advice, pharmacology ☐<br>Other (specify): _____________
Q52.2	Which health facility / office? Select from the list.	*See list*
Q52.3	First and Last name of contact person	
Q52.4	Role of contact person in the facility / office	HF incharge ☐<br>MD ☐<br>Clinician ☐<br>Nurse ☐<br>Midwife ☐<br>Clinical assistant ☐<br>Other medical staff ☐<br>Administrative staff ☐<br>DMO office ☐<br>Specify role: ________
Q52.5	Relationship with the contact person	Only work-related ☐<br>Friend ☐<br>Family ☐

		Former colleague ☐<br>Schoolmate ☐
Q52.6	How did you get in contact with the contact person	In person ☐<br>Phonecall ☐<br>Text message ☐<br>Email ☐<br>Regular mail ☐<br>Other (specify) __________<br>______________________________
Q52.7	How do you evaluate the success of the specific referral, on a scale from 1 to 4 where 1 is failure and 4 is complete success?	Failure Complete success<br>☐ 1 ☐ 2 ☐ 3 ☐ 4
Q53	Referral or advice for clinical or pharmacological matters related to the diagnosis or treatment of a patient affected by "**Cancer**" in the last 3 months?<br><br>If yes, for the last event:	No ☐<br>Yes, diagnosis ☐<br>Yes, treatment ☐
Q53.1	Type of interaction	Referral, clinical matter ☐<br>Referral, pharmacology ☐<br>Advice, clinical matter ☐<br>Advice, pharmacology ☐<br>Other (specify): ______________
Q53.2	Which health facility / office? Select from the list.	*See list*
Q53.3	First and Last name of contact person	
Q53.4	Role of contact person in the facility / office	HF incharge ☐<br>MD ☐<br>Clinician ☐<br>Nurse ☐<br>Midwife ☐<br>Clinical assistant ☐<br>Other medical staff ☐<br>Administrative staff ☐<br>DMO office ☐<br>Specify role: _________
Q53.5	Relationship with the contact person	Only work-related ☐<br>Friend ☐<br>Family ☐<br>Former colleague ☐<br>Schoolmate ☐

Q53.6	How did you get in contact with the contact person	In person ☐<br>Phonecall ☐<br>Text message ☐<br>Email ☐<br>Instant messaging ☐<br>Regular mail ☐<br>Other (specify) __________<br>______________________________
Q53.7	How do you evaluate the success of the specific referral, on a scale from 1 to 4 where 1 is failure and 4 is complete success?	Failure Complete success<br>☐ 1 ☐ 2 ☐ 3 ☐ 4
Q54	Referral or advice for clinical or pharmacological matters related to the diagnosis or treatment of a patient affected by a "**Cardiovascular disease**" in the last 3 months?<br><br>If yes, for the last event:	No ☐<br>Yes, diagnosis ☐<br>Yes, treatment ☐
Q54.1	Type of interaction	Referral, clinical matter ☐<br>Referral, pharmacology ☐<br>Advice, clinical matter ☐<br>Advice, pharmacology ☐<br>Other (specify): ______________
Q54.2	Which health facility / office? Select from the list.	*See list*
Q54.3	First and Last name of contact person	
Q54.4	Role of contact person in the facility / office	HF incharge ☐<br>MD ☐<br>Clinician ☐<br>Nurse ☐<br>Midwife ☐<br>Clinical assistant ☐<br>Other medical staff ☐<br>Administrative staff ☐<br>DMO office ☐<br>Specify role: _________
Q54.5	Relationship with the contact person	Only work-related ☐<br>Friend ☐<br>Family ☐<br>Former colleague ☐<br>Schoolmate ☐
Q54.6	How did you get in contact with the contact person	In person ☐<br>Phonecall ☐<br>Text message ☐<br>Email ☐<br>Regular mail ☐<br>Other (specify) __________<br>______________________________

Q54.7	How do you evaluate the success of the specific referral, on a scale from 1 to 4 where 1 is failure and 4 is complete success?	Failure Complete success<br>□ 1 □ 2 □ 3 □ 4
Q55	Referral or advice for clinical or pharmacological matters related to the diagnosis or treatment of a patient affected by a "**Chronic respiratory disease**" in the last 3 months?<br><br>If yes, for the last event:	No □<br>Yes, diagnosis □<br>Yes, treatment □
Q55.1	Type of interaction	Referral, clinical matter □<br>Referral, pharmacology □<br>Advice, clinical matter □<br>Advice, pharmacology □<br>Other (specify): _____________
Q55.2	Which health facility / office? Select from the list.	*See list*
Q55.3	First and Last name of contact person	
Q55.4	Role of contact person in the facility / office	HF incharge □<br>MD □<br>Clinician □<br>Nurse □<br>Midwife □<br>Clinical assistant □<br>Other medical staff □<br>Administrative staff □<br>DMO office □<br>Specify role: ________
Q55.5	Relationship with the contact person	Only work-related □<br>Friend □<br>Family □<br>Former colleague □<br>Schoolmate □
Q55.6	How did you get in contact with the contact person	In person □<br>Phonecall □<br>Text message □<br>Email □<br>Regular mail □<br>Other (specify) __________
Q55.7	How do you evaluate the success of the specific referral, on a scale from 1 to 4 where 1 is failure and 4 is complete success?	Failure Complete success<br>□ 1 □ 2 □ 3 □ 4
Q56	Referral or advice for clinical or pharmacological matters related to the diagnosis or treatment of a patient affected by a "**Mental illness**" in the last 3 months?<br><br>If yes, for the last event:	No □<br>Yes, diagnosis □<br>Yes, treatment □

Q56.1	Type of interaction	Referral, clinical matter ☐<br>Referral, pharmacology ☐<br>Advice, clinical matter ☐<br>Advice, pharmacology ☐<br>Other (specify): ____________
Q56.2	Which health facility / office? Select from the list.	*See list*
Q56.3	First and Last name of contact person	
Q56.4	Role of contact person in the facility / office	HF incharge ☐<br>MD ☐<br>Clinician ☐<br>Nurse ☐<br>Midwife ☐<br>Clinical assistant ☐<br>Other medical staff ☐<br>Administrative staff ☐<br>DMO office ☐<br>Specify role: ________
Q56.5	Relationship with the contact person	Only work-related ☐<br>Friend ☐<br>Family ☐<br>Former colleague ☐<br>Schoolmate ☐
Q56.6	How did you get in contact with the contact person	In person ☐<br>Phonecall ☐<br>Text message ☐<br>Email ☐<br>Regular mail ☐<br>Other (specify) ________<br>____________________
Q56.7	How do you evaluate the success of the specific referral, on a scale from 1 to 4 where 1 is failure and 4 is complete success?	Failure ... Complete success<br>☐ 1 ☐ 2 ☐ 3 ☐ 4

Section 4: Relational data: administrative issues

Characteristics of the respondent

Q57	First Name and Last Name	
Q58	Date of birth	
Q59	Place of birth (Village, Ward, District, Region)	
Q60	Role in the facility / office	HF incharge □<br>MD □<br>Clinician □<br>Nurse □<br>Midwife □<br>Clinical assistant □<br>Other medical staff □<br>Administrative staff □<br>Department head □<br>Specify role: ________
Q61	For hospitals and health centers, in which department are you working?	
Q62	How long have you worked at this health facility?	Years _______ Months________
Q63	Last highest educational title	
Q64	Place where last education was completed (District, Region)	
Q65	Number of employees dealing with administrative issues	
Q66	Number of members in the HFGC	
Q67	Is there a cascade coaching system in place for medicine management?	No □<br>Yes □
Q68	What is the name of reference facility for cascade supervision	*See list*
Q69	What is the name of the person in charge of cascade coaching?	
Q70	Advice, request or authorization with regards to "**Budgeting for investments in the facility**" in the last year.<br><br>If yes, for the last event:	No □<br>Yes, advice □<br>Yes, request □<br>Yes, authorization □
Q70.1	Which health facility / office? Select from the list.	*See list*
Q70.2	First and Last name of contact person	
Q70.3	Role of contact operator	HF incharge □<br>MD □<br>Clinician □<br>Nurse □<br>Midwife □<br>Clinical assistant □<br>Other medical staff □<br>Administrative staff □<br>DMO office □<br>Specify role: ________

Q70.4	Relationship with the contact person	Only work-related □<br>Friend □<br>Family □<br>Former colleague □<br>Schoolmate □
Q70..5	How did you get in contact with the contact person	In person □<br>Phonecall □<br>Text message □<br>Email □<br>Regular mail □<br>Other (specify) __________<br>______________________________
Q70.6	How do you evaluate the success of the specific contact, on a scale from 1 to 4 where 1 is failure and 4 is complete success?	Failure Complete success<br>□ □ □ □<br>1 2 3 4
Q71	Advice, request or authorization with regards to "**Allocation or management of human resources**" in the last year.<br><br>If yes, for the last event:	No □<br>Yes, advice □<br>Yes, request □<br>Yes, authorization □
Q71.1	Which health facility / office?	*See list*
Q71.2	First and Last name of contact person	
Q71.3	Role of contact operator	HF incharge □<br>MD □<br>Clinician □<br>Nurse □<br>Midwife □<br>Clinical assistant □<br>Other medical staff □<br>Administrative staff □<br>DMO office □<br>Specify role: _________
Q71.4	Relationship with the contact person	Only work-related □<br>Friend □<br>Family □<br>Former colleague □<br>Schoolmate □
Q71.5	How did you get in contact with the contact person	In person □<br>Phonecall □<br>Text message □<br>Email □<br>Regular mail □<br>Other (specify) __________<br>______________________________

Q71.6	How do you evaluate the success of the specific contact, on a scale from 1 to 4 where 1 is failure and 4 is complete success?	Failure Complete success<br>□ 1 □ 2 □ 3 □ 4
Q72	Advice, request or authorization with regards to "**Drug management**" in the last year.<br><br>If yes, for the last event:	No □<br>Yes, advice □<br>Yes, request □<br>Yes, authorization □
Q72.1	Which health facility / office? Select from the list.	*See list*
Q72.2	First and Last name of contact person	
Q72.3	Role of contact operator	HF incharge □<br>MD □<br>Clinician □<br>Nurse □<br>Midwife □<br>Clinical assistant □<br>Other medical staff □<br>Administrative staff □<br>DMO office □<br>Specify role: ________
Q72.4	Relationship with the contact person	Only work-related □<br>Friend □<br>Family □<br>Former colleague □<br>Schoolmate □
Q72.5	How did you get in contact with the contact person	In person □<br>Phonecall □<br>Text message □<br>Email □<br>Regular mail □<br>Other (specify) __________<br>______________________________
Q72.6	How do you evaluate the success of the specific contact, on a scale from 1 to 4 where 1 is failure and 4 is complete success?	Failure Complete success<br>□ 1 □ 2 □ 3 □ 4
Q73	Advice, request or complaint with regards to "**Leakage of funds or corruption-related offenses that took place at the health facility**" in the last year.<br><br>If yes, for the last event:	No □<br>Yes, advice □<br>Yes, request □<br>Yes, complaint □
Q73.1	Which health facility / office? Select from the list.	*See list*
Q73.2	First and Last name of contact person	

Q73.3	Role of contact operator	HF incharge ☐<br>MD ☐<br>Clinician ☐<br>Nurse ☐<br>Midwife ☐<br>Clinical assistant ☐<br>Other medical staff ☐<br>Administrative staff ☐<br>DMO office ☐<br>Specify role: ________
Q73.4	Relationship with the contact person	Only work-related ☐<br>Friend ☐<br>Family ☐<br>Former colleague ☐<br>Schoolmate ☐
Q73.5	How did you get in contact with the contact person	In person ☐<br>Phonecall ☐<br>Text message ☐<br>Email ☐<br>Regular mail ☐<br>Other (specify) __________<br>______________________________
Q73.6	How do you evaluate the success of the specific contact, on a scale from 1 to 4 where 1 is failure and 4 is complete success?	Failure / Complete success<br>☐ 1 ☐ 2 ☐ 3 ☐ 4

A.4 Density of responses for specific categories of care, by district

Question	Kilolo DC (Iringa) N=42	Msalala DC (Shinyanga) N=27
**Child care**		
Emergency conditions	12 referrals	10 referrals
Diagnostic approaches	9 referrals	11 referrals
Cough or difficulty breathing	1 referrals	12 referrals
Diarrhoea	1 referrals	6 referrals
Fever	9 referrals	17 referrals
Severe acute malnutrition	12 referrals	14 referrals
Complications related to HIV/AIDS	5 referrals	11 referrals
Surgical problems	0 referrals	1 referral
Supportive care	2 referrals	7 referrals
**Treatment of NCD's**		
Diabetes	13 referrals	4 referrals
Cancer	1 referrals	5 referrals
Cardiovascular disease	29 referrals	10 referrals
Chronic respiratory disease	2 referrals	15 referrals
Mental illness	3 referrals	1 referral

A.5 Diagnostic and goodness of fit outputs for Exponential Random Graph Models (ERGMs)

A.5.1 Diagnostics ERGM for Kilolo, Childcare, model 1

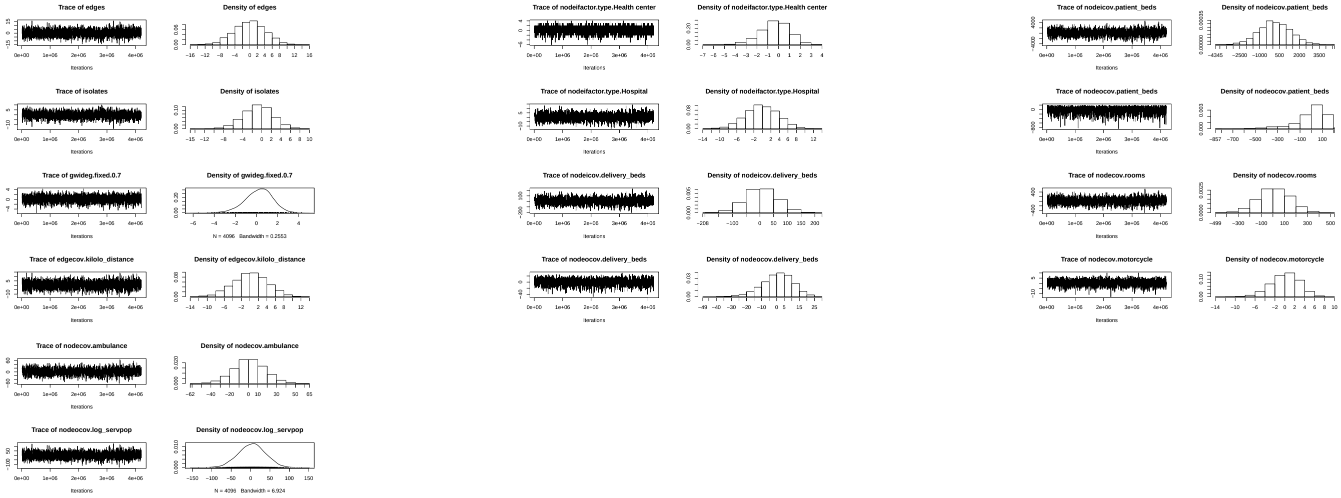

A.5.2 Diagnostics ERGM for Kilolo, Childcare, model 2

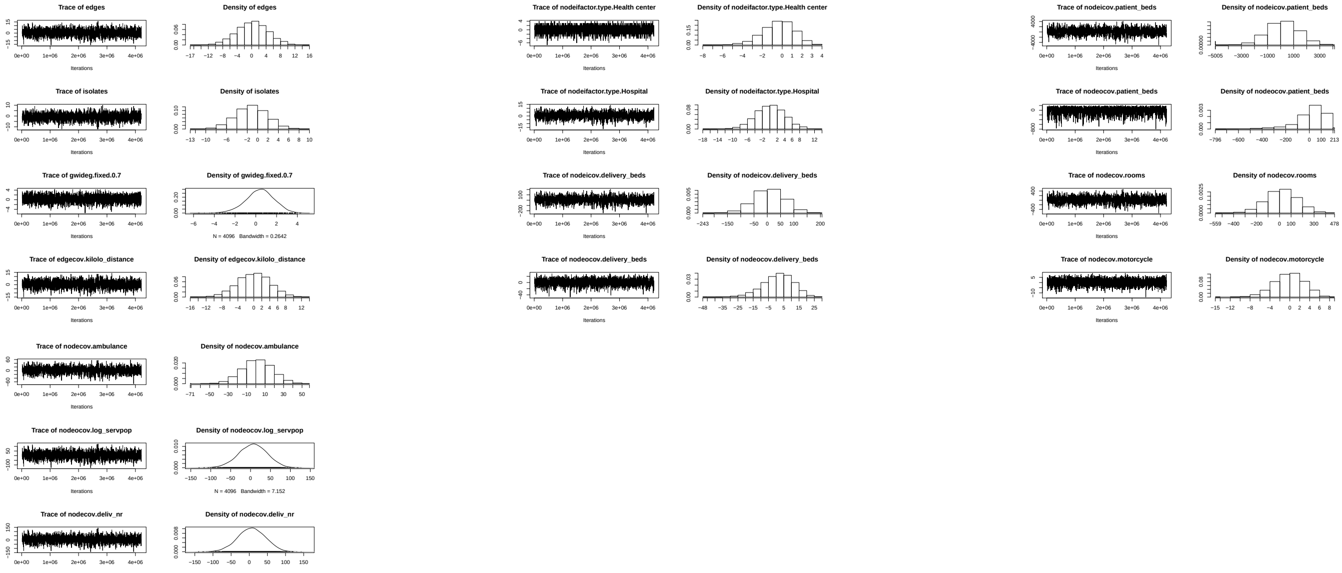

A.5.3 Diagnostics ERGM for Kilolo, Treatment of NCDs

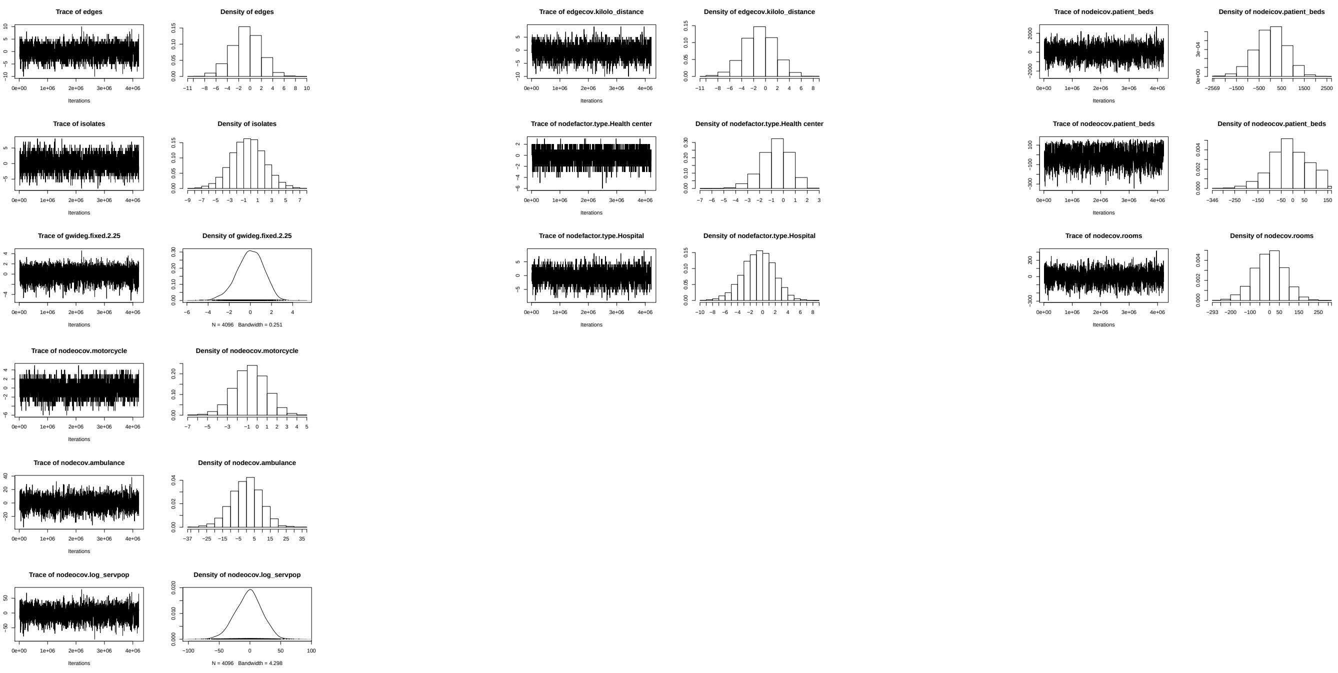

A.5.4 Diagnostics ERGM for Msalala, Childcare, model 1

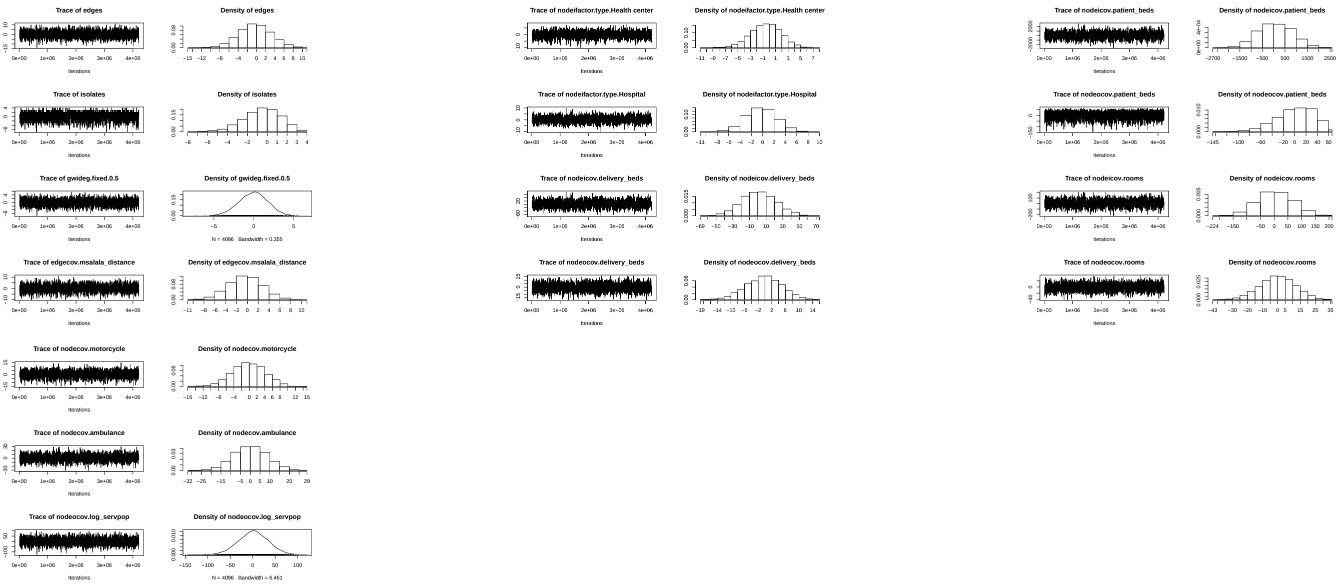

A.5.5 Diagnostics ERGM for Msalala, Childcare, model 2

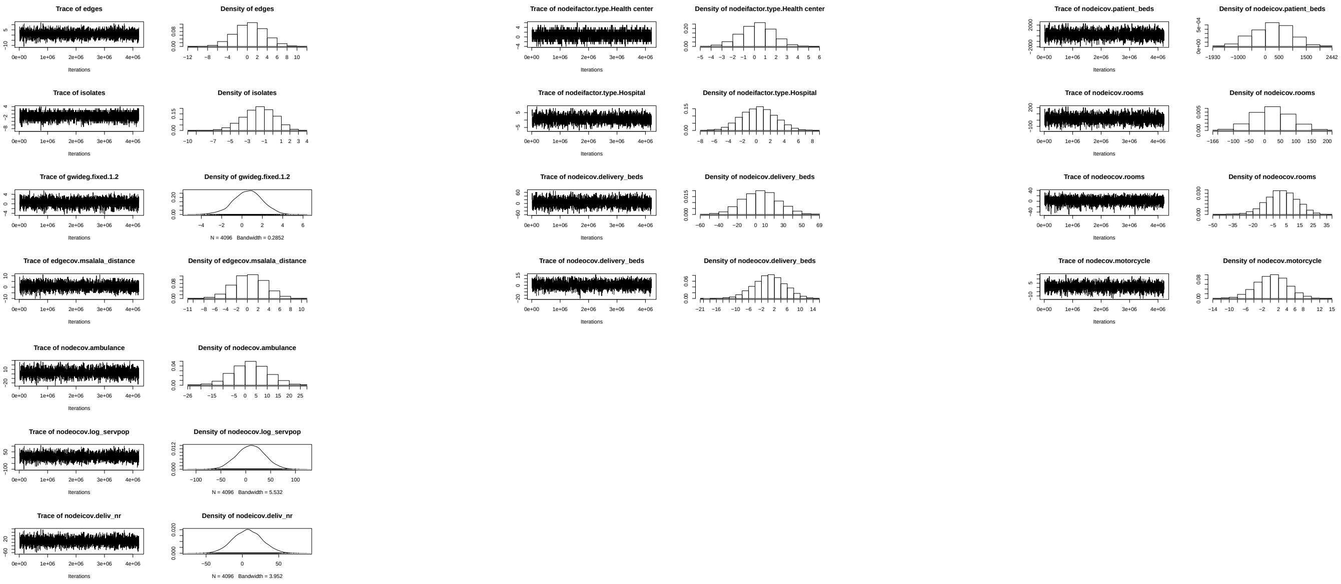

A.5.6 Diagnostics ERGM for Msalala, Treatment of NCDs

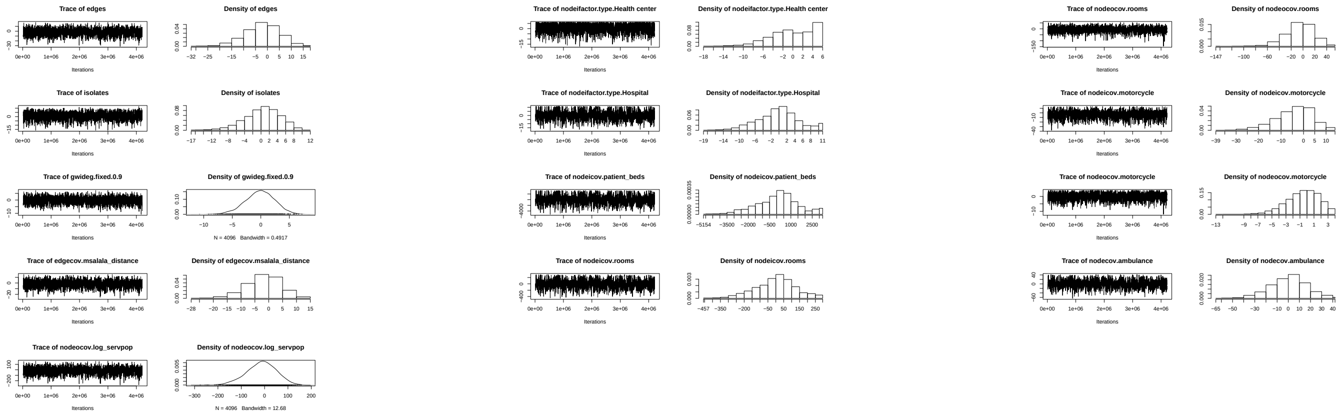

A.5.7 GOF ERGM for Kilolo, Childcare, model 1

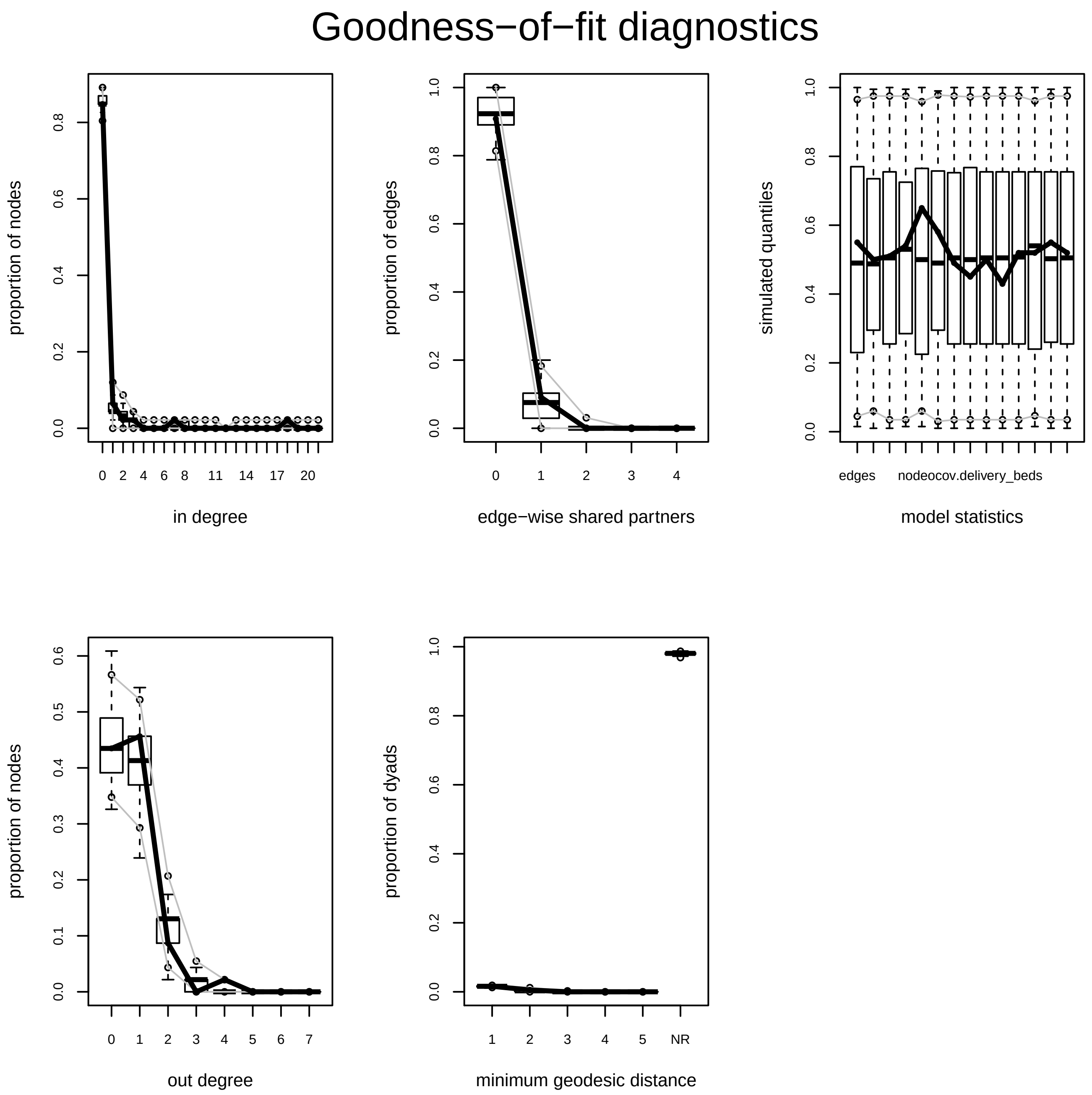

A.5.8 GOF ERGM for Kilolo, Childcare, model 2

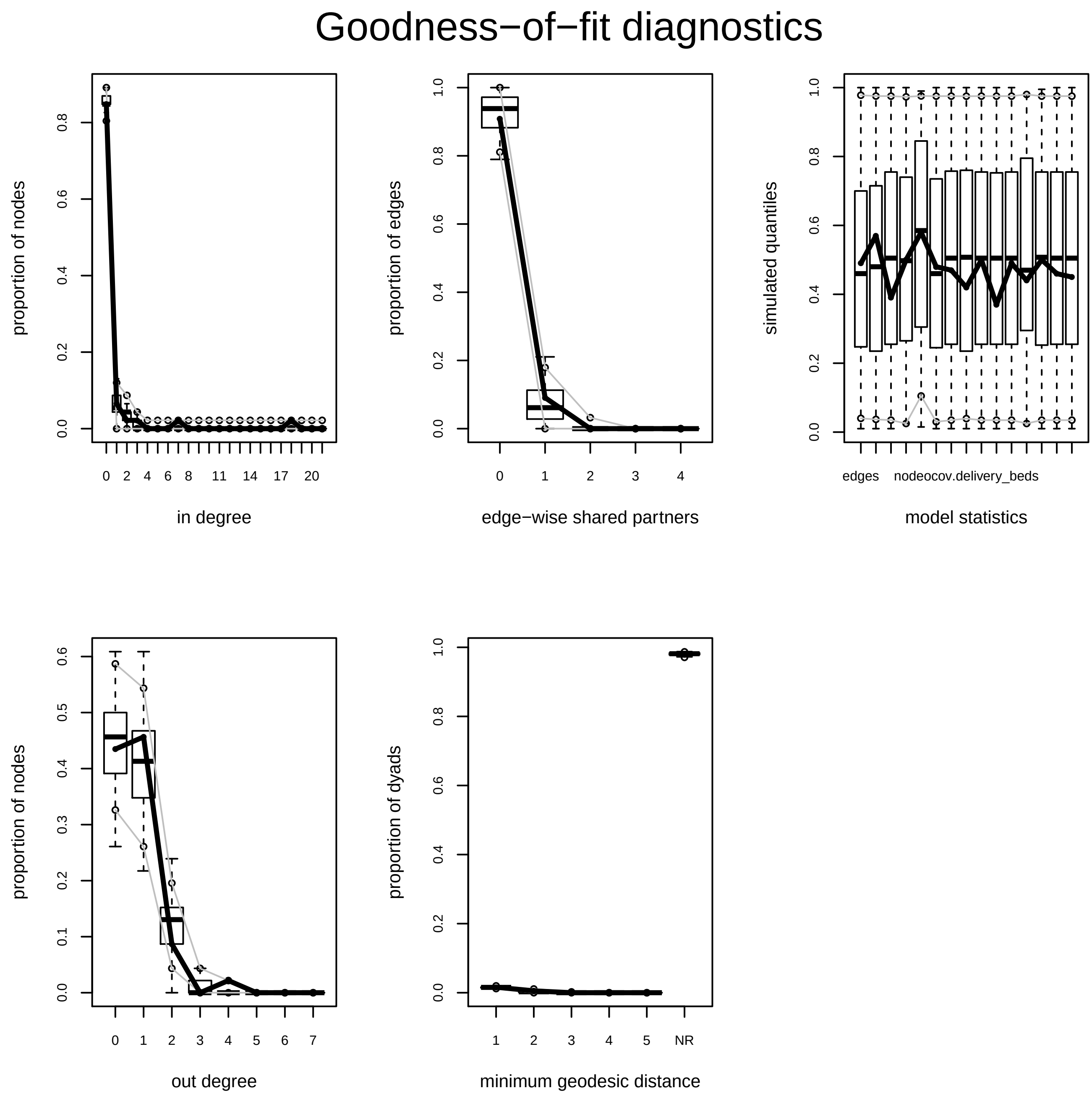

A.5.9 GOF ERGM for Kilolo, Treatment of NCDs

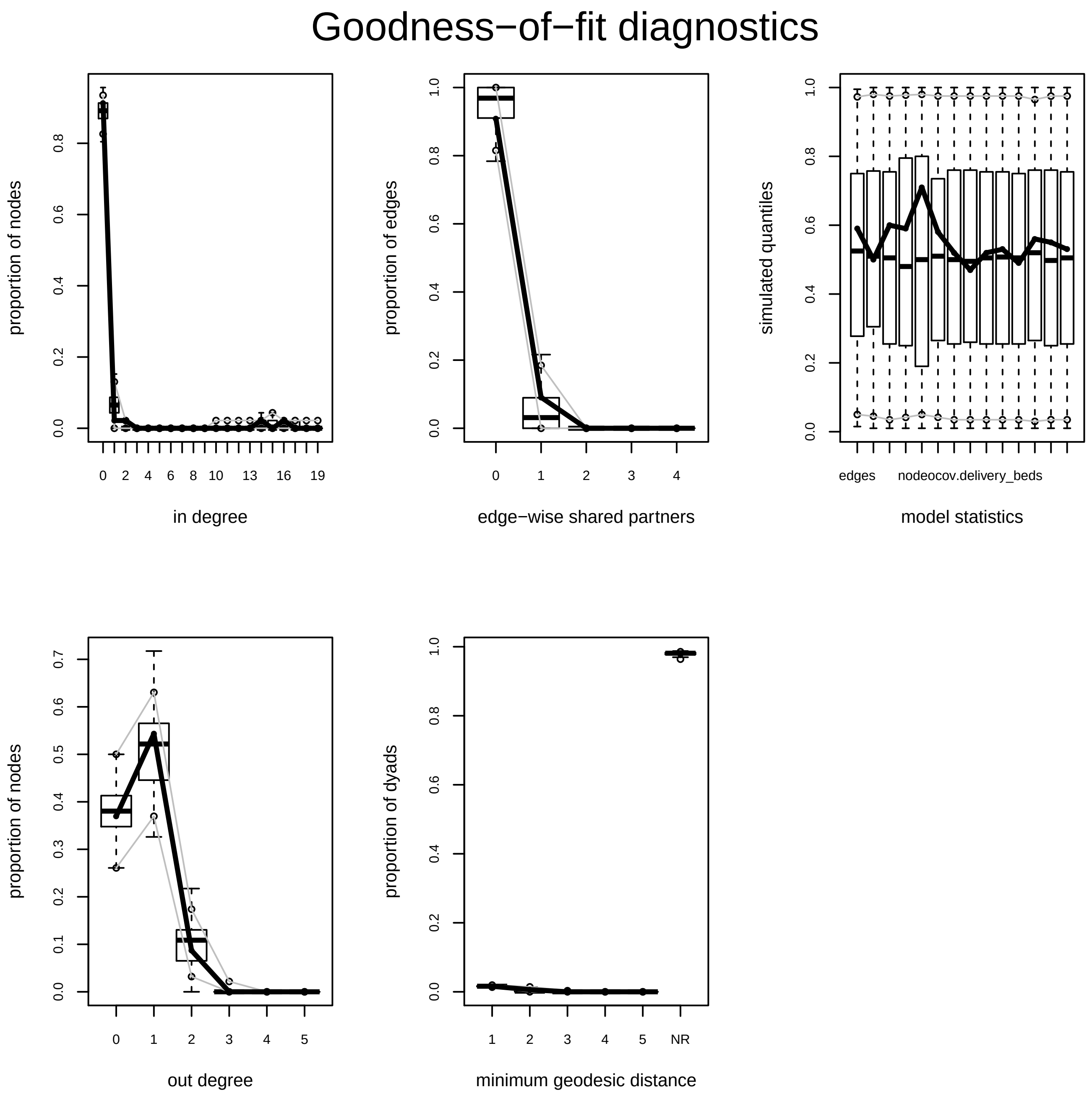

A.5.10 GOF ERGM for Msalala, Childcare, model 1

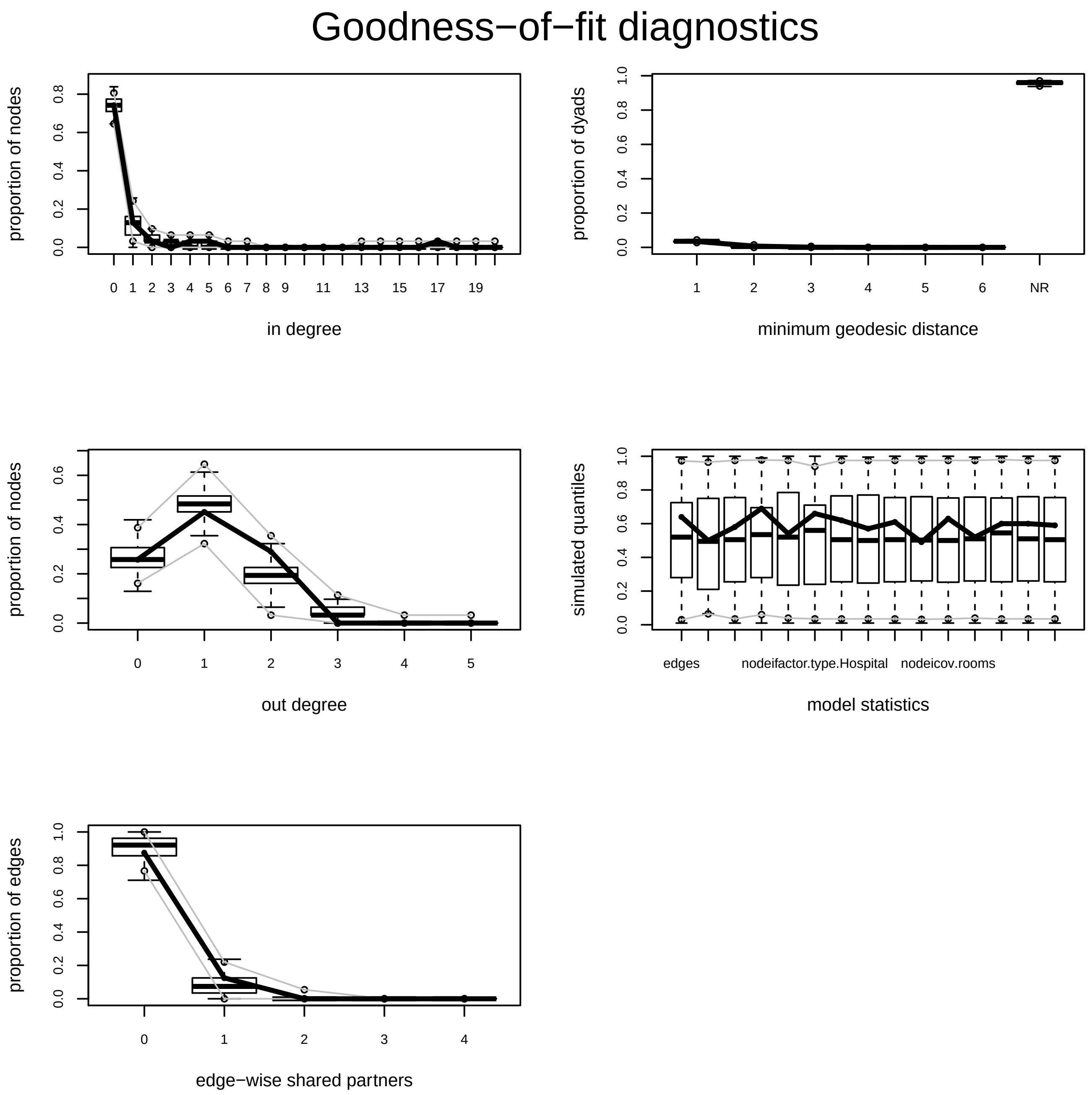

A.5.11 GOF ERGM for Msalala, Childcare, model 2

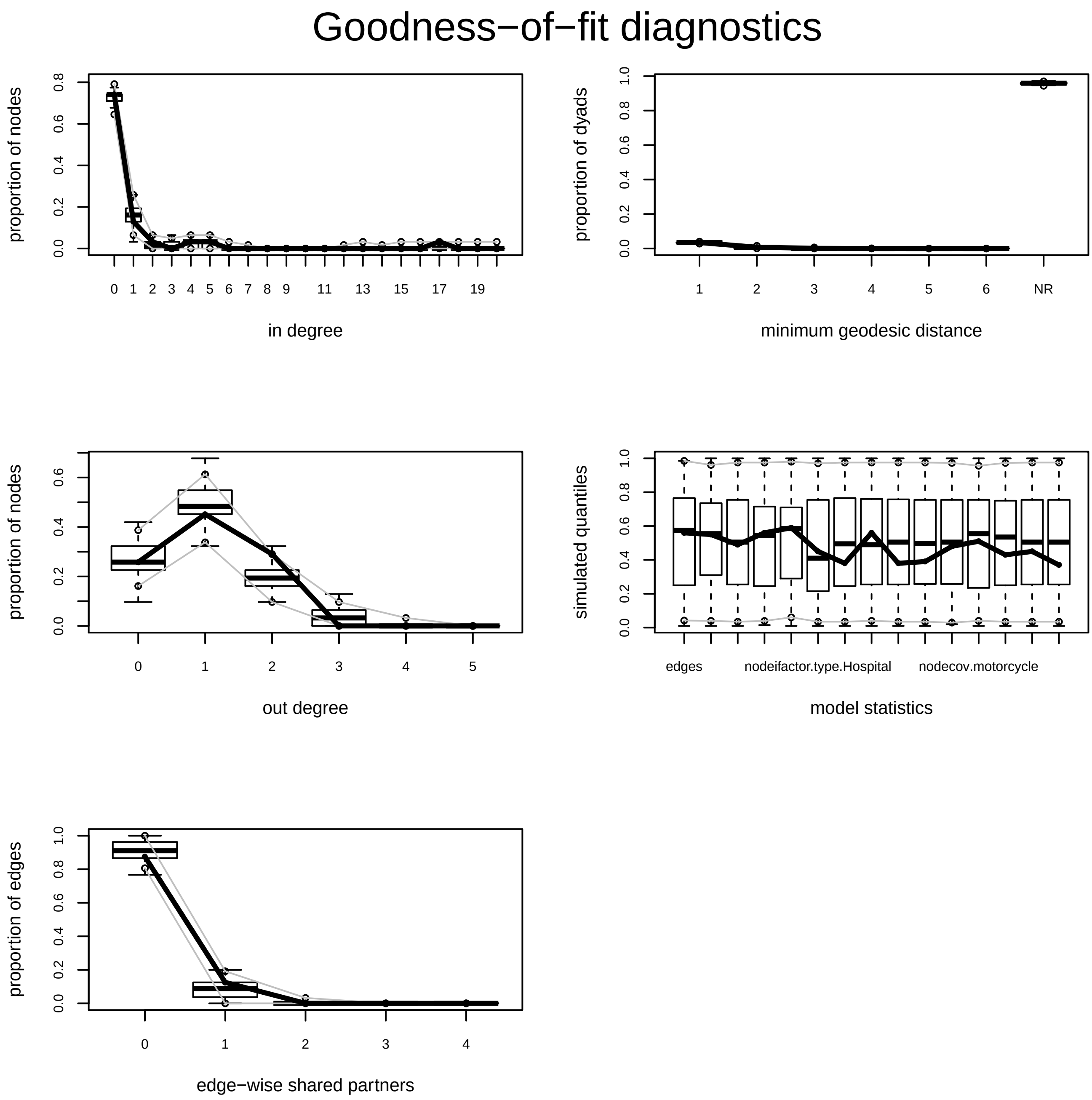

A.5.12 GOF ERGM for Msalala, Treatment of NCDs

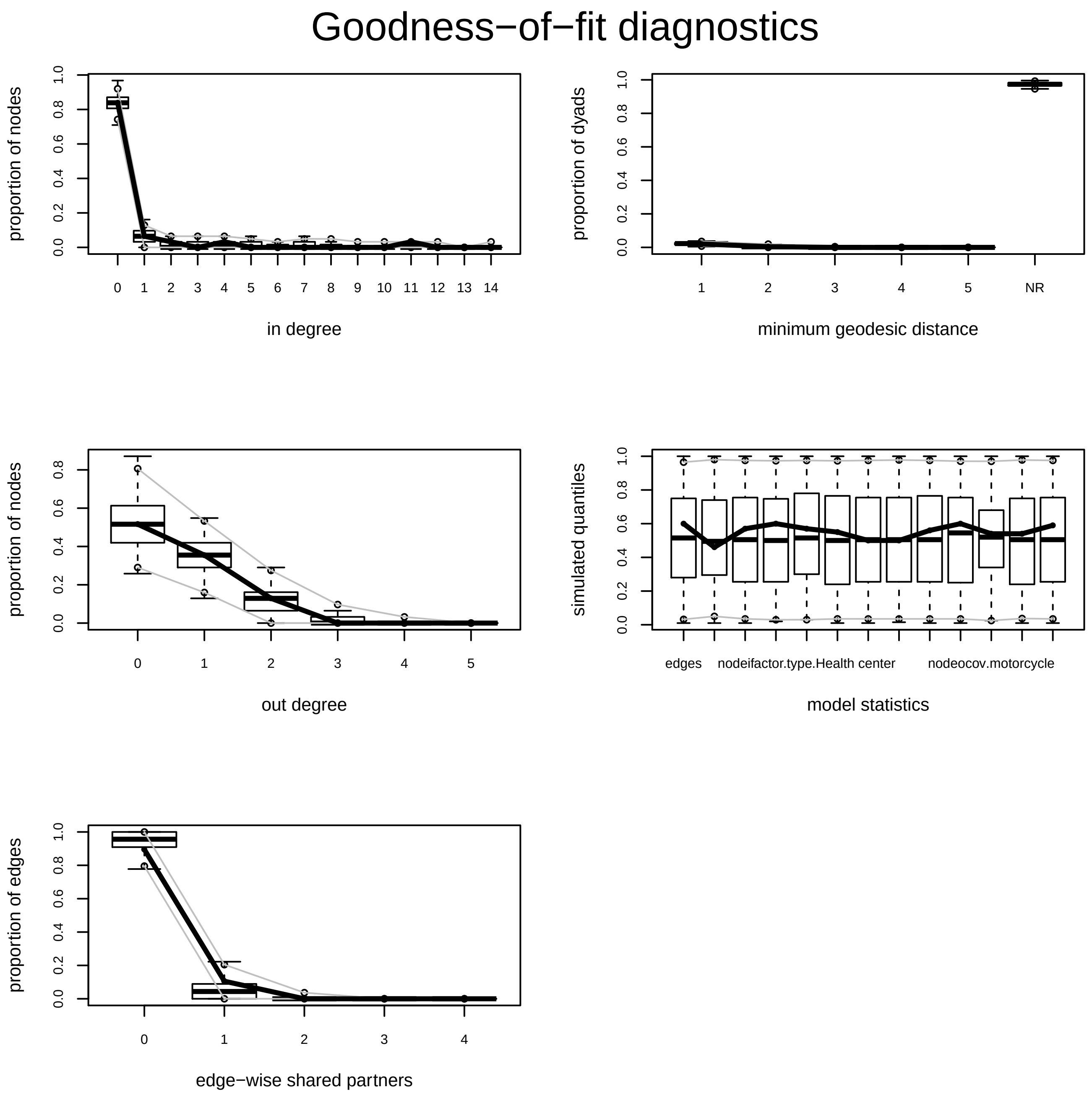

A.6 Interpretation of ERGM coefficients: simple numerical example

Tables 3 and 4 illustrate the features of our referral networks. The network of referrals for treatment of NCDs in Kilolo has 46 nodes (facilities) and 33 observed edges (referrals). Thus, the potential number of directed edges is

$$N \times (N - 1) = 46 \times 45 = 2070$$

Accordingly, the density of the network is

$$\frac{33}{2070} = 0.015942$$

In an ERGM framework, modelling network formation only as a function of the number of edges would result in the following output.

	Model 1
edges	-4.12^{***}
	(0.18)
AIC	340.63
BIC	346.27
Log Likelihood	-169.32

$^{***}p < 0.001$; $^{**}p < 0.01$; $^{*}p < 0.05$

This model assumes that ties appear randomly between pairs given the number of existing edges and nodes. The coefficient associated to edges is significant and equal to -4.12. If we apply the inverse-logit function

$$L^{-1}(\alpha) = \frac{e^{\alpha}}{1 + e^{\alpha}}$$

to the coefficient for "edges" above, we obtain exactly 0.015942, which is the value for density.
