# Supplementary material for: A network analysis of patient referrals in two district health systems in Tanzania
Source: Health Policy Plan. 2020 Dec 24;36(2):162–75. doi: 10.1093/heapol/czaa138 (PMC7996649; doi:10.1093/heapol/czaa138)
Supplement: czaa138_Supplementary_Data [file czaa138_supplementary_data.zip › 20200904_table1.docx]

Table 1: Descriptive statistics about districts.

|  | **Kilolo DC** | **Msalala DC** |
| --- | --- | --- |
| Region | Iringa region | Shinyanga region |
| Country zone | Southern highlands | Lake, Northwest |
| District population (2019) | 262’431 | 323’587 |
| Region population estimate (2019) | 1’149’481 | 1’993’589 |
| Region population density (pers./km2) | 26 | 81 |
| Life expectancy at birth (region, years) | 44 | 55 |
| Malaria mortality (region, per 100’000) | 21.38 | 28.67 |
| Under 5 mortality rate (region, per 1’000) | 145.1 | 104.3 |
| Facility deliveries (% of total, 2019) | 55.8 | 78.1 |
| Share of children with reported birth weight below 2.5kg (2019) | 6.5 | 5.9 |
| Share of caesarean section deliveries (2019) | 14.1 | 2.0 |
| Share of children with pentavalent vaccine at 1 year | 89.2 | 88.6 |
| Full availability of 10 tracer medicines (% of facilities) | 96.0 | 96.5 |
| Health workers density (per 10’000, 2018) | 6.3 | 3.9 |
| Dispensaries |  |  |
| Public | 40 | 24 |
| Faith-based | 15 | 2 |
| Private | 5 | 3 |
| Health centres |  |  |
| Public | 1 | 3 |
| Faith-based | 1 | - |
| Private | - | 1 |
| Hospitals |  |  |
| Public | - | - |
| Faith-based | 1 (district designated) | - |
| Private | - | - |
| HPSS project | No | Yes, since 2015 |

Notes: (1) Data from National Bureau of Statistics (2013), MoHCDGEC, NBS, OCGS and ICF International (2016), Malaria Atlas Project (2017) and MOHCDGEC (2020); (2) The list of 10 tracer medicines considered includes: disposable syringe and needles, oral rehydration salts, albendazole/mebendazole oral, amoxycillin/cotrimoxazole, artemether/lumefantrine oral, depo provera, supplies for malaria microscopy, saline solution/dextrose, pentavalent vaccine, oxytocin/ergometrine/misoprostol; (3) Health workers density related to public facilities for the following cadres: nurse, clinical assistant, clinical officer, medical officer, pharmacist, nursing officer; (4) Msalala district has no public district hospital. The closest district referral hospital is in the neighbouring district of Kahama.
