# Supplementary material for: A network analysis of patient referrals in two district health systems in Tanzania
Source: Health Policy Plan. 2020 Dec 24;36(2):162–75. doi: 10.1093/heapol/czaa138 (PMC7996649; doi:10.1093/heapol/czaa138)
Supplement: czaa138_Supplementary_Data [file czaa138_supplementary_data.zip › 20200904_table2.docx]

Table 2: Characteristics of the surveyed sample of facilities and respondents.

|  | **Kilolo DC** | **Msalala DC** |
| --- | --- | --- |
| Facilities *N (%)* |  |  |
| Dispensary | 40 (95.2) | 24 (88.9) |
| Health centre | 1 (2.4) | 3 (11.1) |
| Hospital | 1 (2.4) | 0 (0.0) |
| Total staff assigned (*mean, median, range)* | 5.97, 3, 1-109 | 6.52, 5, 1-28 |
| Respondent qualification *N (%)* |  |  |
| Health facility in-charge (MD, clinician, nurse) | 16 (38.1) | 8 (29.6) |
| Clinician or clinical assistant (not in-charge) | 3 (7.1) | 3 (11.1) |
| Nurse (not in-charge) | 19 (45.2) | 15 (55.6) |
| Midwife (not in-charge) | 4 (9.5) | 1 (3.7) |
| Years of tenure *(mean, median, range)* | 5.21, 3.37, 0.3-30 | 3.20, 3, 0.5-10 |
