# Supplementary material for: A network analysis of patient referrals in two district health systems in Tanzania
Source: Health Policy Plan. 2020 Dec 24;36(2):162–75. doi: 10.1093/heapol/czaa138 (PMC7996649; doi:10.1093/heapol/czaa138)
Supplement: czaa138_Supplementary_Data [file czaa138_supplementary_data.zip › 20200904_table3.docx]

Table 3: Descriptive statistics for health facilities in the networks.

|  | **Kilolo DC**  *Mean, Median, Range* | **Msalala DC**  *Mean, Median, Range* |
| --- | --- | --- |
| Total *(N)* | 46 | 31 |
| Dispensary | 40 | 25 |
| Health center | 3 | 4 |
| Hospital | 3 | 2 |
| Private health facilities *(N)* | 0 | 1 |
| Faith-based facilities *(N)* | 3 | 1 |
| Population served | 29111, 3321, 1200-950000 | 64471, 10995, 610-1535000 |
| Rooms in the building/compound | 5.17, 4, 1-36 | 4.61, 3, 1-26 |
| Patient beds | 21.72, 3, 0-366 | 24.35, 2, 0-300 |
| Delivery beds | 2.022, 1, 0-17 | 1.64, 1, 0-8 |
| ICU beds | 0, 0, 0 | 0, 0, 0 |
| Nr. of ambulance vehicles | 0.26, 0, 0-5 | 0.35, 0, 0-3 |
| Nr. of motorcycles | 0.26, 0, 0-2 | 0.26, 0, 0-2 |
| Facility deliveries in the 3 months prior to the survey | 65.98,8.5,1-1159 | 125.2,19,5-1892 |
| Facility deliveries  (per 1’000 people served) | 9.82, 3.02, 0.40-272.97 | 7.35, 2.73,0.32-52.00 |

Source: MOHCDGEC (2020); Sawe et al., (2014).
