# Supplementary material for: A network analysis of patient referrals in two district health systems in Tanzania
Source: Health Policy Plan. 2020 Dec 24;36(2):162–75. doi: 10.1093/heapol/czaa138 (PMC7996649; doi:10.1093/heapol/czaa138)
Supplement: czaa138_Supplementary_Data [file czaa138_supplementary_data.zip › 20200904_table4.docx]

Table 4: Descriptive statistics for networks of referrals related to treatment of childhood illnesses and NCDs.

|  | **Kilolo DC** | **Msalala MC** |
| --- | --- | --- |
| *Treatment of childhood illnesses* | | |
| Number of referrals | 33 | 32 |
| Referrals to private or faith-based facilities | 9 | 1 |
| Referrals outside of district boundaries | 21 | 19 |
| Referrals |  |  |
| Between dispensaries | - | 3 |
| From dispensaries to health centres | 5 | 10 |
| From dispensaries to hospitals | 22 | 16 |
| From health centres to dispensaries | 1 | - |
| Between health centres | - | 1 |
| From health centres to hospitals | 2 | 2 |
| From district hospital to regional hospital | 3 | - |
| Network density | 0.016 | 0.034 |
| Referrals per 10’000 outpatient visits | 7.34 | 8.25 |
| Referrals per 1’000 RCH visits | 24.00 | 13.69 |
| *Treatment of NCDs* | | |
| Number of referrals | 33 | 19 |
| Referrals to private or faith-based facilities | 15 | 1 |
| Referrals outside of district boundaries | 17 | 12 |
| Referrals |  |  |
| Between dispensaries | 0 | 2 |
| From dispensaries to health centres | 2 | 6 |
| From dispensaries to hospitals | 25 | 8 |
| Between health centre | - | - |
| From health centres to hospitals | 1 | 3 |
| From district hospital to regional hospital | 5 | - |
| Network density | 0.016 | 0.020 |
| Referrals per 10’000 outpatient visits | 7.34 | 4.89 |

*Note: Ratio of inpatient, outpatient and Reproductive and Child Health (RCH) visits based on reported number of visits at the surveyed (referring) health facilities in the 3 months prior to the survey date.*
