# Supplementary material for: A network analysis of patient referrals in two district health systems in Tanzania
Source: Health Policy Plan. 2020 Dec 24;36(2):162–75. doi: 10.1093/heapol/czaa138 (PMC7996649; doi:10.1093/heapol/czaa138)
Supplement: czaa138_Supplementary_Data [file czaa138_supplementary_data.zip › 20200904_table5.docx]

Table 5: Facilities with highest In-degree and betweenness scores for referrals in Kilolo and Msalala districts.

| **Kilolo DC** | | | | | | | |
| --- | --- | --- | --- | --- | --- | --- | --- |
| *Treatment of childhood illnesses* | | | | *Treatment of NCDs* | | | |
| *In-degree* | *Value* | *Betweenness* | *Value* | *In-degree* | *Value* | *Betweenness* | *Value* |
| Regional  hospital | 18 | Ililula  hospital | 6 | Regional  hospital | 16 | Ililula  hospital | 13 |
| Ililula  hospital | 7 | Kibadaga health centre | 5 | Ililula  hospital | 14 | - | - |
| Kidabaga  health centre | 2 | - | - | Kidabaga health centre | 2 | - | - |
| **Msalala DC** | | | | | | | |
| *Treatment of childhood illnesses* | | | | *Treatment of NCDs* | | | |
| *In-degree* | *Value* | *Betweenness* | *Value* | *In-degree* | *Value* | *Betweenness* | *Value* |
| Kahama  hospital | 17 | Lunguya  health centre | 4 | Kahama  hospital | 11 | Chela health centre | 4 |
| Chela  health centre | 5 | Segese  dispensary | 2 | Chela health centre | 4 | - | - |
| Lunguya  health centre | 4 | Bugarama  health centre | 2 | Lunguya health centre | 2 | - | - |
