# Supplementary material for: A network analysis of patient referrals in two district health systems in Tanzania
Source: Health Policy Plan. 2020 Dec 24;36(2):162–75. doi: 10.1093/heapol/czaa138 (PMC7996649; doi:10.1093/heapol/czaa138)
Supplement: czaa138_Supplementary_Data [file czaa138_supplementary_data.zip › 20200904_table6.docx]

Table 6: Exponential random graph models for referral networks related to treatment of childhood illnesses

|  | Kilolo DC | | Msalala MC | |
| --- | --- | --- | --- | --- |
|  | Model 1 | Model 2 | Model 1 | Model 2 |
| Edges | -11.62* (4.69) | -10.70* (4.16) | -3.78 (2.66) | -24.06*** (7.20) |
| Isolates | -0.06 (0.61) | -0.11 (0.61) | -0.83 (0.77) | -0.67 (0.77) |
| Geometrically weighted in-degree distribution | 4.61 (3.23) | 3.82 (2.67) | -0.58 (1.61) | 10.54** (3.89) |
| Edge covariate: road distance (KM) | -1.78** (0.64) | -1.86** (0.65) | -3.74*** (0.72) | -3.57*** (0.72) |
| Incoming ties, node factor: health centre | 6.52** (2.22) | 5.70** (1.93) | 4.05* (1.90) | 11.86** (2.78) |
| Incoming ties, node factor: hospital | 10.46** (3.66) | 9.62** (3.41) | -2.02 (9.81) | 19.78*** (3.64) |
| Incoming ties, node covariate: delivery beds | -0.24 (0.56) | -0.39 (0.54) | 1.19** (0.45) | 2.21* (0.92) |
| Outgoing ties, node covariate: delivery beds | 0.27 (0.28) | 0.19 (0.30) | 0.39 (0.64) | 0.68 (0.66) |
| Incoming ties, node covariate: patient beds | -0.01 (0.02) | -0.01 (0.01) | 0.04 (0.04) | 0.10 (0.07) |
| Outgoing ties, node covariate: patient beds | -0.04 (0.02) | -0.04 (0.02) | -0.05 (0.04) |  |
| Incoming ties, node covariate: nr. of rooms |  |  | -0.15 (0.29) | -2.90*** (1.03) |
| Outgoing ties, node covariate: nr. of rooms |  |  | -0.20 (0.25) | -0.35 (0.20) |
| Combined node covariate: nr. of rooms | 0.30 (0.16) | 0.32* (0.16) |  |  |
| Node covariate: number of motorcycles | -0.31 (0.56) | -0.41 (0.56) | 0.07 (0.62) | -0.43 (0.66) |
| Node covariate: number of ambulances | 0.13 (0.66) | 0.12 (0.63) | 0.34 (0.73) | -0.01 (0.80) |
| Node covariate: log of catchment population | -0.14 (0.36) | -0.25 (0.38) | -0.01 (0.27) | -0.07 (0.26) |
| Incoming ties, log number of facility deliveries |  | 0.22 (0.27) |  | 4.46*** (1.38) |
| GWIDEG decay parameter | 0.7 | 0.7 | 0.5 | 1.2 |
| AIC | 186.67 | 188.03 | 145.18 | 132.97 |
| BIC | 265.56 | 272.56 | 2217.70 | 205.50 |
| Log Likelihood | -79.33 | -79.01 | -57.59 | -51.48 |
| *Notes: Coefficients represent contributions to log-odds. Standard errors in parentheses. *** p < 0.001, ** p < 0.01, * p < 0.05* | | | | |
