# Supplementary material for: A network analysis of patient referrals in two district health systems in Tanzania
Source: Health Policy Plan. 2020 Dec 24;36(2):162–75. doi: 10.1093/heapol/czaa138 (PMC7996649; doi:10.1093/heapol/czaa138)
Supplement: czaa138_Supplementary_Data [file czaa138_supplementary_data.zip › 20200904_table7.docx]

Table 7: Exponential random graph models for networks of referrals related to treatment of NCDs

|  | Kilolo DC | Msalala MC |
| --- | --- | --- |
| Edges | 34.97*** (9.47) | -0.68 (1.89) |
| Isolates | -1.69* (0.76) | 0.07 (0.60) |
| Geometrically weighted in-degree distribution | 9.83 (4.19) | -3.19*** (0.85) |
| Edge covariate: geographic distance (KM) | -2.33*** (0.76) | -1.74* (0.74) |
| Incoming ties, node factor: health centre |  | 2.31** (0.85) |
| Incoming ties, node factor: hospital |  | 6.07*** (0.31) |
| Combined node covariate: health centre | 3.84** (1.40) |  |
| Combined node covariate: hospital | 15.71*** (4.04) |  |
| Incoming ties, node covariate: patient beds | -0.07*** (0.02) | 0.01 (0.02) |
| Outgoing ties, node covariate: patient beds | -0.25*** (0.06) |  |
| Incoming ties, node covariate: nr. of rooms |  | -0.27  (0.18) |
| Outgoing ties, node covariate: nr. of rooms |  | -0.13 (0.14) |
| Combined node covariate: nr. of rooms | 0.65** (0.22) |  |
| Incoming ties, node covariate: nr. of motorcycles |  | 0.96* (0.40) |
| Outgoing ties, node covariate: nr. of motorcycles | 0.21 (0.69) | -0.04 (0.61) |
| Node covariate: number of ambulances | 1.02 (0.94) | 0.07 (0.71) |
| Outgoing ties, node covariate: log of catchment population | 1.54** (0.60) | -0.04 (0.22) |
| GWIDEG decay parameter | 2.25 | 0.9 |
| AIC | 141.24 | 133.58 |
| BIC | 208.87 | 196.44 |
| Log Likelihood | -58.62 | -53.79 |
| *Notes: Coefficients represent contributions to log-odds. Standard errors in parentheses.  *** p < 0.001, ** p < 0.01, * p < 0.05* | | |
